# Rating norms should be calculated from cumulative link mixed effects models

**DOI:** 10.3758/s13428-022-01814-7

**Published:** 2022-09-14

**Authors:** Jack E. Taylor, Guillaume A. Rousselet, Christoph Scheepers, Sara C. Sereno

**Affiliations:** School of Psychology and Neuroscience, University of Glasgow, 62 Hillhead Street, Glasgow, G12 8QB UK

**Keywords:** Norms, Ratings, Concreteness, Character similarity

## Abstract

Studies which provide norms of Likert ratings typically report per-item summary statistics. Traditionally, these summary statistics comprise the mean and the standard deviation (SD) of the ratings, and the number of observations. Such summary statistics can preserve the rank order of items, but provide distorted estimates of the relative distances between items because of the ordinal nature of Likert ratings. Inter-item relations in such ordinal scales can be more appropriately modelled by cumulative link mixed effects models (CLMMs). In a series of simulations, and with a reanalysis of an existing rating norms dataset, we show that CLMMs can be used to more accurately norm items, and can provide summary statistics analogous to the traditionally reported means and SDs, but which are disentangled from participants’ response biases. CLMMs can be applied to solve important statistical issues that exist for more traditional analyses of rating norms.

**Supplementary Information:**

The online version contains supplementary material available at 10.3758/s13428-022-01814-7.

## Introduction

In a typical norming study, participants are asked to rate features of stimuli on Likert scales (e.g., on a scale from 1 to 7). These ratings are used to estimate how participants perceive these features. Such estimates may be used to validate stimuli for an existing experiment, design new stimuli, or correlate with observations of behaviour or neural activity. For the latter two purposes, the estimates are often made public for use by other researchers alongside dedicated publications. Examples include but are not limited to ratings on various dimensions, for stimuli as diverse as words (Brysbaert et al., 2014; Scott et al., 2019; Warriner et al., 2013), orthographic characters (Simpson et al., 2013), photographs of objects (Brodeur et al., 2014) or faces (Ma et al., 2015), and melodies (Belfi & Kacirek, 2021). Such norming studies are typically summarised via per-item statistics of means and standard deviations (SDs) of the ratings for each item. In this article, we argue that ordinal models can provide more robust measures of item norms. We focus on cumulative link mixed effects models (CLMMs), showing that they can yield summary statistics analogous to the traditional estimates of means and SDs, but disentangled from artefacts of nonlinearities in participants’ response patterns.

Datasets of norms typically report, for each individual item, the mean of the Likert ratings, the SD of the Likert ratings, and the number of observations. These reflect, respectively, estimated values, variability in these values, and sample size from which the summary statistics are calculated. These simple metrics are intuitive and easy to calculate, and can be used to rank items on the rated dimension. However, the use of means and SDs to accurately estimate distances between normed items would require that Likert scales are continuous, with an equal step size between each successive option. In fact, while the dimension participants are judging may scale continuously when measured objectively (e.g., age of acquisition), and while a Likert scale may be presented to participants with equal steps between options (e.g., via radio button inputs), there is no reason to assume that *judgements* on the target dimension are graduated linearly. Instead, Likert scales are examples of ordinal scales, with responses scaling in one direction (i.e., 1<2<3<4<5…), but not necessarily in equal steps. At the very least, the true relationship between ratings and the dimension(s) they are supposed to measure remains underspecified. By norming items on an ordinal variable via their means and SDs, researchers produce estimates which can be distorted by nonlinearities in the scaling of Likert judgements (Liddell & Kruschke, 2018). If researchers were only interested in ranking items, summaries like the mean would be sufficient. However, it is often useful to accurately know the relative distances between items in the target dimension. For instance, item norms are frequently included in statistical analyses as continuous variables or predictors (e.g., Fernandino et al., 2016; Goh et al., 2016; Hollis & Westbury, 2016; Khanna & Cortese, 2021; Perry et al., 2018; Pexman et al., 2019; Scott et al., 2019; Vejdemo & Hörberg, 2016). We note that instances where researchers dichotomise a rated feature to compare the *N* highest- and lowest-rated items may be less impacted by distortions in averages of Likert ratings, as the comparison is still essentially ordinal. However, such dichotomisation will result in an unnecessary loss of statistical power and precision if continuous alternatives are available (MacCallum et al., 2002; Royston et al., 2006).

**Fig. 1:**
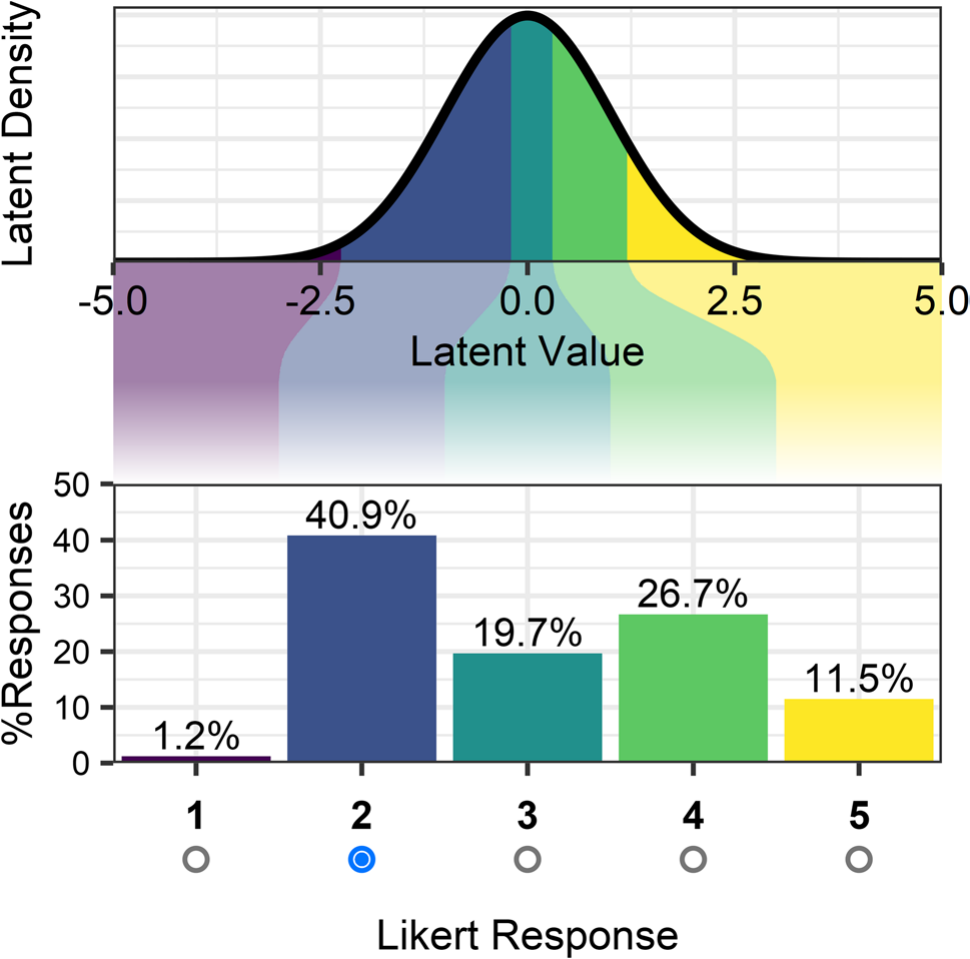
The assumed relationship between a continuous latent distribution and ordinal Likert responses (here, on a 1–5 scale). Each Likert response corresponds to a region of the latent distribution, highlighted in corresponding colours. The probability of observing any given Likert response is the probability of a value being drawn from the latent distribution which is between the lower and upper bounds of that Likert response’s region. In the example illustrated, the latent distribution is assumed to be normal (as is the case for a probit-link function). The nonlinearities in this example response pattern mean that the most likely response would be *2*, while the responses of *1* and *5* would be comparably rare. This response pattern would therefore bias means of observations towards the Likert response *2*, and away from the scale’s extremities

An alternative approach to summarising Likert judgements is to assume that a latent continuous distribution *underlies* the ordinal scale, allowing any given ordinal response to be converted into possible latent values (Fig. 1). This is the approach implemented in cumulative link models (CLMs), where ordinal dependent variables are mapped onto ordered regions of a latent distribution (Bürkner & Vuorre, 2019; McCullagh, 1980). Responses are commonly modelled via probit- or logit-link functions which, respectively, assume that the latent variable is normally or logistically distributed. The model estimates the locations of ordered thresholds demarcating the borders between regions of the latent distribution associated with each response, while other coefficients can estimate a constant shift in the location of the distribution associated with changes in the values of predictors (i.e., slopes). The CLM approach can be extended to account for *multilevel data* in the form of CLMMs, which allow the researcher to estimate not only the values of population-level intercepts and slopes (i.e., fixed effects), but also how these intercepts and slopes differ across members of distinct populations which are sampled in the data (i.e., random, or “varying”, effects). For instance, a CLMM can estimate how the mean latent value associated with each individual participant or item differs from that of the population average.

The need for ordinal models such as CLMs and CLMMs to appropriately model ordinal responses is already commonly recognised in the analysis of experiments (Liddell & Kruschke, 2018). Correspondingly, several tools currently exist, and are already widely used, to fit CLMMs, such as the *ordinal* (Christensen, 2020) package for the R programming language (R Core Team, 2021). When these models are applied, however, they are typically used to estimate the effects of experimental manipulations (i.e., fixed effects); when CLMMs are applied, random effects are typically included to account for participant and item variability, thereby improving accuracy of fixed effect estimation, but are rarely examined in any detail beyond a cursory glance at summary statistics like random effects variances. Estimating a CLMM with by-item random effects could, however, also be used to norm items in a manner which is not distorted by participants’ response patterns. Indeed, random effects in such models are per-unit (per-item, per-participant, etc.) estimates of each unit’s most likely deviation from the corresponding fixed effect, in link units. CLMMs and related ordinal models assume the overall mean of the latent distribution (i.e., what would be the fixed-effect intercept in a linear model) to be equal to zero, for identifiability. In the case of a CLMM with per-item random effects, therefore, the extracted random effects will represent estimates of the latent mean associated with each item. In R, these values are stored within a fitted CLMM object, and can be extracted, for example, via the generic R function ranef(). Norming items via random effects in this way confers additional benefits, such as improvements in accuracy associated with shrinkage (where outlying, unlikely values, are appropriately pulled towards more likely estimates) and the concurrent estimation of additional sources of variability (such as per-participant random effects). In this article, we argue that CLMMs are well suited to calculating norms from Likert responses, and solve key issues associated with more traditional analyses of norming studies.

**Fig. 2:**
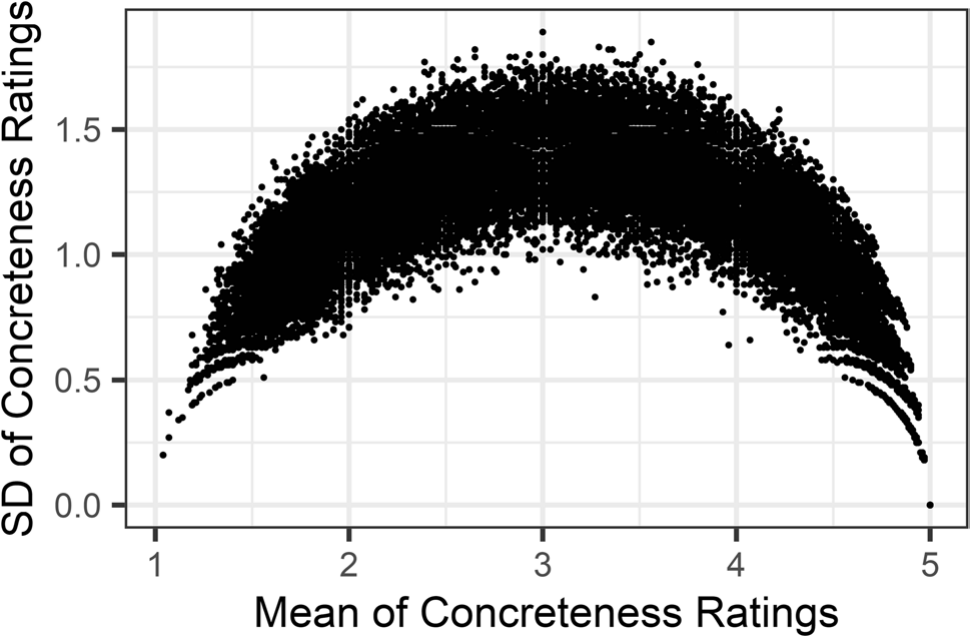
The relationship between the mean and SD of items’ Likert ratings (1–5 scale) in word concreteness, from Brysbaert et al. (2014). The pattern suggests that responses are most consistent at the extremes of the Likert scale, but that items with averages at the midpoints of the Likert scale elicit less consistent responses

One issue with traditional analyses of norming studies centres around the finding that heterogenous relationships are frequently observed between means and SDs. Notably, Pollock (2018) highlighted a common relationship in ratings of word concreteness (Fig. 2), whereby the lowest SDs are observed at the extremes of a Likert scale, while items towards the centre of the scale show much higher SDs. Such heterogeneity should be expected to some degree for any scale which has lower and upper bounds. However, Pollock showed that although it was common for participants to agree on ratings at the extremes of the scale (*1* and *5)*, such inter-rater reliability was exceedingly rare for ratings at midpoints in the scale. Pollock interpreted this finding as evidence that participants’ judgements on dimensions showing this overall pattern are largely dichotomous. It was argued that Likert scales are inappropriate for norming items on variables with dichotomous responses, and that averages at the centre of the scale merely reflect polarisation in responses, rather than a meaningful estimate. For instance, if half of all responses for a single item were *1*, and half were *5*, this would result in an average Likert response of *3*, even though no participant gave this response. This inconsistency would also be reflected by a high SD of ≥ 2.

Pollock’s argument has been criticised by Neath and Surprenant (2020), who examined whether a concreteness effect in a serial word recall task differs between words with low or high SDs in Likert judgements. If mid-scale responses are less meaningful, they may be expected to predict effects of concreteness less well. Neath and Suprenant showed, however, that the effect of word concreteness was estimated as a consistent effect size when average Likert responses are used as the predictor, regardless of how large the SDs of Likert ratings are for the presented items. Further to this, we argue that Pollock’s interpretation of the mean–SD relationship suggests that Likert responses are expected to be continuous, rather than ordinal. When Likert responses are instead viewed as ordinal regions of a latent continuous variable, a unimodal latent distribution can lead to an apparent dichotomy in Likert responses, and responses can appear inconsistent even when there are meaningful differences in the latent distribution. Such a pattern could arise from any response pattern where the lowest and highest Likert responses (e.g., *1* and *5* on a five-point scale) account for large portions of the latent distribution, increasing the likelihood of any given latent value being mapped onto an extreme Likert response, while responses at the scale’s centre (e.g., *2*, *3*, and *4*), account for much less, making these mid-scale responses comparably less likely. Importantly, even though responses could *appear* dichotomous in such cases, changes in the relative likelihood of the different Likert responses would still track meaningful shifts in the central tendency of the latent distribution. Furthermore, lower SDs at the extremes of a scale may reflect floor and ceiling effects, rather than agreement among raters. There may be meaningful differences between items that share the minimum or maximum possible average rating, which are nonetheless undetectable within the limited bounds of the rating scale.

**Fig. 3:**
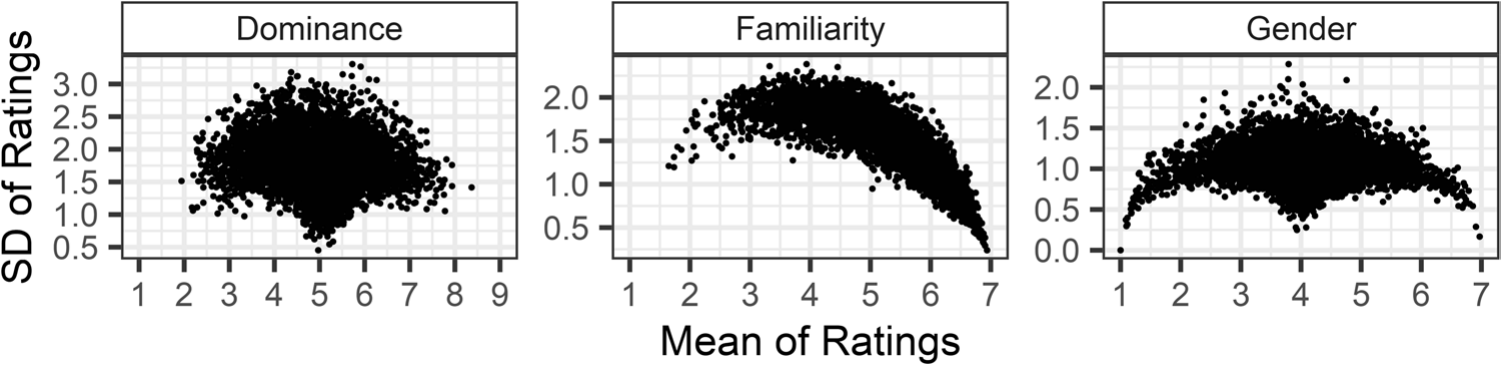
Mean–SD relationships for judgements of words on three semantic variables in the Glasgow Norms (Scott et al., 2019). Dominance was judged on a 1–9 Likert scale, while Familiarity and Gender were judged on 1–7 scales

When dichotomous response patterns are explained with reference to ordered regions of a latent distribution, it is clear that many other response patterns should also be possible, and that these would result in distinct patterns in the mean–SD relationship of Likert responses (Brainerd et al., 2021). In any pattern, items whose average is closer to regions that participants are biased towards should be more likely to show greater consistency in responses, and thus have lower SDs, while items further from these regions will be more likely to have higher SDs. Figure 3 shows the mean–SD relationships observed in the Likert judgements of words on three different semantic variables from the Glasgow Norms (Scott et al., 2019): Dominance, Familiarity, and Gender. Each of these variables shows a qualitatively different mean–SD relationship distinct from that identified by Pollock (2018). For Dominance, the lowest and highest SDs, respectively reflecting the greatest and least consistency, are at the centre of the Likert scale, and no items are observed at or close to the scale endpoints. This suggests that, for this sample, judgements of words’ dominance are biased towards a mid-point response or are dichotomous, and that there was never any consensus among raters for items having extreme Dominance values. For Familiarity, in contrast, responses are most consistent at the upper end of the Likert scale, with lower SDs observed as average Familiarity increases. Further, the average Likert response never reaches lower than 1.5, suggesting that for this sample of items participants rarely consistently agree that a word is unfamiliar. In the case of Gender, three separate regions of the Likert scale show the lowest SDs, with intervening responses never showing such consistency. These three regions may suggest that participants were biased towards three different responses: *1* for highly male, *4* for gender neutral, and *7* for highly female. It is important to note, however, that the highest SDs are also observed at the gender-neutral centre of the scale, suggesting that the average Likert response for some words may index polarisation in responses, with dichotomous ratings as either highly male or highly female. An example of such a word is *bridegroom*, a compound word which technically refers to a man, yet consists of two highly, yet oppositely, gendered words, *bride* and *groom*. The inconsistency observed for words like *bridegroom* stands in contrast to the consistent gender neutrality observed for words whose Gender ratings also average to 4, but which result in low SDs (e.g., the words *impaired*, *name*, *occurrence*). This highlights that variance in Likert judgements can reflect meaningful differences, such as an item’s ambiguity or discriminability. If overall variance is calculated on raw Likert responses, however, these meaningful differences in variance will be entangled with differences in response consistency that result from the overall response pattern.

If variance reflects meaningful differences among items, how can it be estimated without distortion from response patterns? One solution could be for researchers to estimate both a latent mean and latent SD for each item that is normed. Although CLMMs traditionally assume homogeneity of variance, the framework provided by CLMMs may be extended to simultaneously describe meaningful differences in both the central tendency and spread of a latent distribution. Just as latent means are analogous to raw means, estimates of latent SDs are analogous to raw SDs, similarly disentangled from response patterns. While most CLMMs, including those fit by the *ordinal* package (Christensen, 2020), exclusively model changes in the central tendency of a latent distribution (assuming homogenous variance across observations), it is possible to fit a model which concurrently describes changes in both the variance and central tendency of the latent distribution. The *brms* package (Bürkner, 2018) for R, an interface to STAN (STAN Development Team, 2021), provides an accessible solution to fit such models. Here, in addition to multi-level changes in the mean of the latent distribution, a discrimination parameter can be estimated, as the inverse of the latent SD (Bürkner & Vuorre, 2019). As the models are estimated via Markov chain Monte Carlo (MCMC) sampling, translating the discrimination parameter of each posterior sample to the SD, before calculating summary statistics, will allow the calculation of random effects for the variance of the latent distribution. CLMMs can therefore provide researchers with analogues to the traditionally reported statistics of means and SDs, but with both estimates disentangled from participants’ response patterns.

We argue that CLMMs provide a valuable framework for norming items via Likert scales, allowing the calculation of items’ *latent* means and SDs, analogous to the traditional estimates of means and SDs of responses, but disentangled from overall response patterns. In the first half of this article, through a series of simulations, we demonstrate the following: (1) non-linear response patterns can account for the typical relationships observed between means and SDs of ratings, and CLMMs can appropriately model items’ values in the latent distribution underlying Likert responses; (2) such models can be expanded to account for other sources of variability, such as participant random effects, with improvements in the accuracy of item estimates; (3) such models can be further expanded to account for differences in a latent distribution’s variance as well as its mean; and (4) while CLMMs make assumptions about the underlying latent distribution, they are relatively robust to modelling responses that result from distributions which violate these assumptions, and are still preferable to the traditional approach of calculating raw means of ordinal responses. In the second half of the article, we apply CLMMs to real data from an existing dataset on judgements of character similarities (Simpson et al., 2013), showing how these methods and results differ from those of traditional analyses.

## Simulations

To demonstrate that CLMMs provide comparable results across different response patterns, we performed simulations as follows. On each iteration, a single dataset is simulated which has differences between observations, items, and (from Simulation 2 onwards) participants, described in terms of the mean and SD of a normally distributed latent distribution. This normal distribution represents latent values before they are distorted by an overall pattern in the Likert responses. Differences between items and participants are similarly drawn from normal distributions – we model these differences via the random-effect structure of the CLMM. The values from this single dataset are then mapped onto one of five possible response patterns. This is done to show (a) how identical effects in latent space can result in divergent estimates and patterns when using traditional means and SDs, and (b) how CLMMs provide estimates which are far less biased by overall response patterns.

**Fig. 4:**
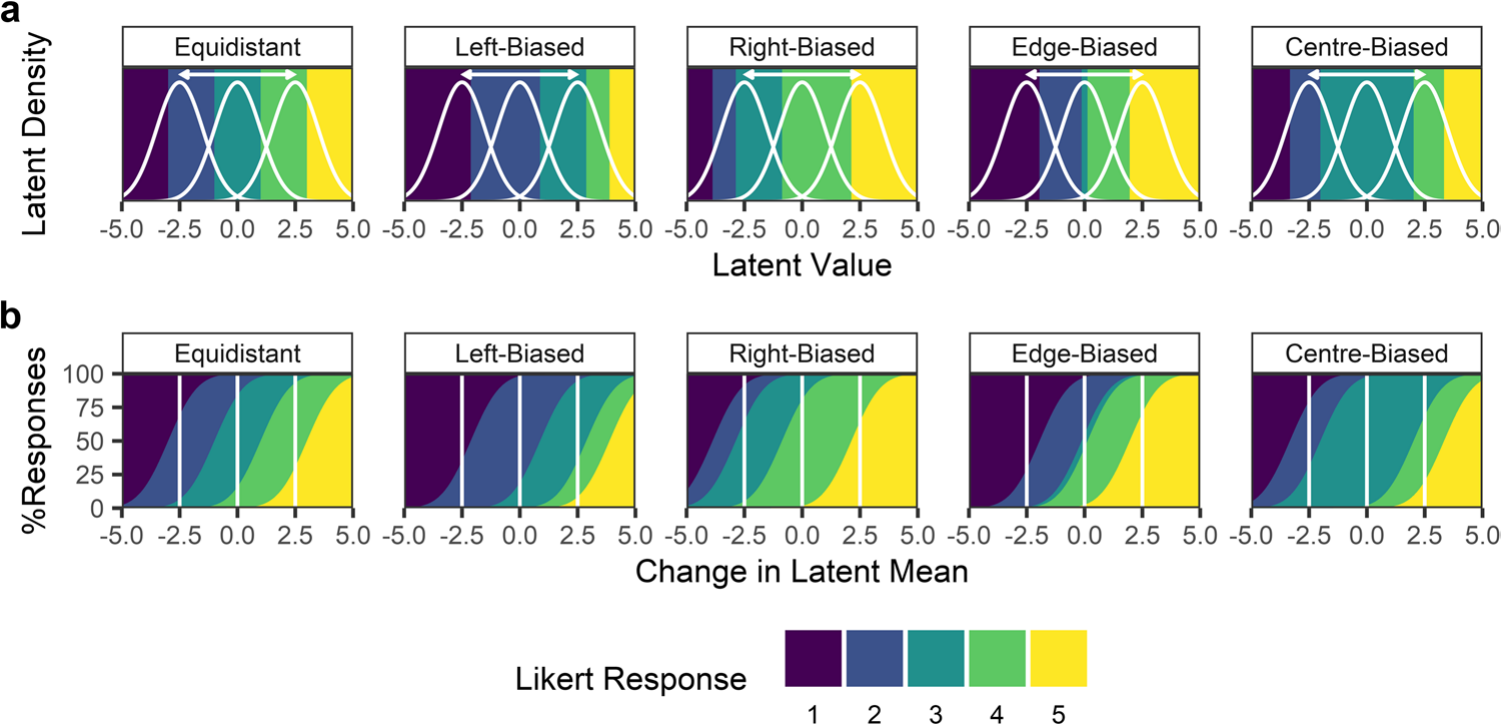
Illustration of how response patterns affect Likert responses. Given the same latent distribution, the five example response patterns we used in the simulations alter the probability of different Likert responses. The top half of the figure, **a**, shows the locations of the thresholds for each response pattern, highlighting how changes in the latent mean alter the proportion of the latent distribution which maps onto each Likert response. Importantly, this effect differs among response patterns. To illustrate this point, for each response pattern, the densities of three example distributions (*white curves*) are shown, with means of –2.5, 0, and 2.5, and an identical SD of 1. An observation sampled from one of these distributions would fall into one coloured region and would be mapped onto the corresponding Likert response. The bottom half of the figure, **b**, shows how the change in the mean of the latent distribution (on the *x*-axis) alters the probability (cumulative percentage; *y*-axis) of observing any Likert response differentially in each of the five response patterns for an identical latent distribution of mean 0 and SD 1. The same example three distributions as in panel **a** are highlighted with *white* *vertical lines*

Throughout the simulations, we use five example response patterns, as follows: equidistant, left-biased, right-biased, edge-biased, and centre-biased. These are similar to the qualitative categories of response styles identified in the item response theory literature (Baumgartner & Steenkamp, 2001). The only difference between the response patterns we simulate is in the locations of the thresholds demarcating the borders between regions of the latent distribution which map onto respective ordinal observations. The differences between the five response patterns are illustrated in Fig. 4, which shows how the probabilities of ordinal Likert responses differ among the response patterns, even when the change in the latent distribution is identical; differences in the probability of each response are accounted for entirely by changes in locations of thresholds.

In each simulation, we map simulated latent values onto a corresponding Likert response according to each response mapping. For example, a latent value of 2.5 on one trial would be recoded to responses *4*, *3*, *5, 5,* and *4* for the equidistant, left-biased, right-biased, edge-biased, and centre-biased response patterns, respectively. In this way, the results across the different response patterns are directly comparable. The only exception to this is Simulation 4, where we manipulated the distribution of latent variables and random effects but kept the response mapping constant. In every simulation, we recovered the item random effect values after fitting a separate CLMM to ratings simulated for each response pattern, using a probit-link function to reflect the normal distribution of the simulated latent distribution. Here, the retrieved item random effects encode the difference between each item and the overall distribution in a parameter describing the latent distribution (usually the latent mean, but in Simulation 3, also the latent SD). For instance, a random effect of – 1.2 for a single item’s latent mean would indicate a shift of the full latent distribution of –1.2 away from the grand mean (which, for CLMs, is always 0). Each CLMM was fit with either the *ordinal* (Christensen, 2020) or *brms* (Bürkner, 2018) package for R.

The code used in all simulations is available at the OSF project associated with this article, at https://osf.io/ntvmf/.

### Simulation 1: CLMMs with item random effects

In this first simulation, we demonstrate that cumulative links appropriately account for nonlinear response patterns, and that random effects can be used to accurately calculate differences in a Likert scale’s underlying latent distribution for separate items. In each iteration, we simulated 100 individual items’ positions on a latent distribution with a mean of 0 and SD of 1. We also simulated residual variance in the latent distribution with a normal distribution of mean 0, and SD 1. As such, latent distribution values for item *i*, *L*_*i*_, were simulated as follows, where *μ*_*i*_ refers to item random effects, and *e*_*i*_ refers to the residuals.

$${\displaystyle \begin{array}{c}{L}_i={\mu}_i+{e}_i\\ {}{\mu}_i\sim N\left(0,1\right)\\ {}{e}_i\sim N\left(0,1\right)\end{array}}$$

In each iteration, we generated 25 latent means for each item, given that item’s random effect *μ*_*i*_, with these values then recoded to ordinal responses on a five-item Likert scale, as described above. To recover (via ranef()) the item random effect values, we used the *ordinal* package (Christensen, 2020) to fit a CLMM to ratings simulated for each response pattern, with a probit-link function. In the package’s syntax, the model was specified as follows:

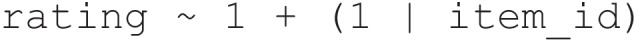



**Fig. 5:**
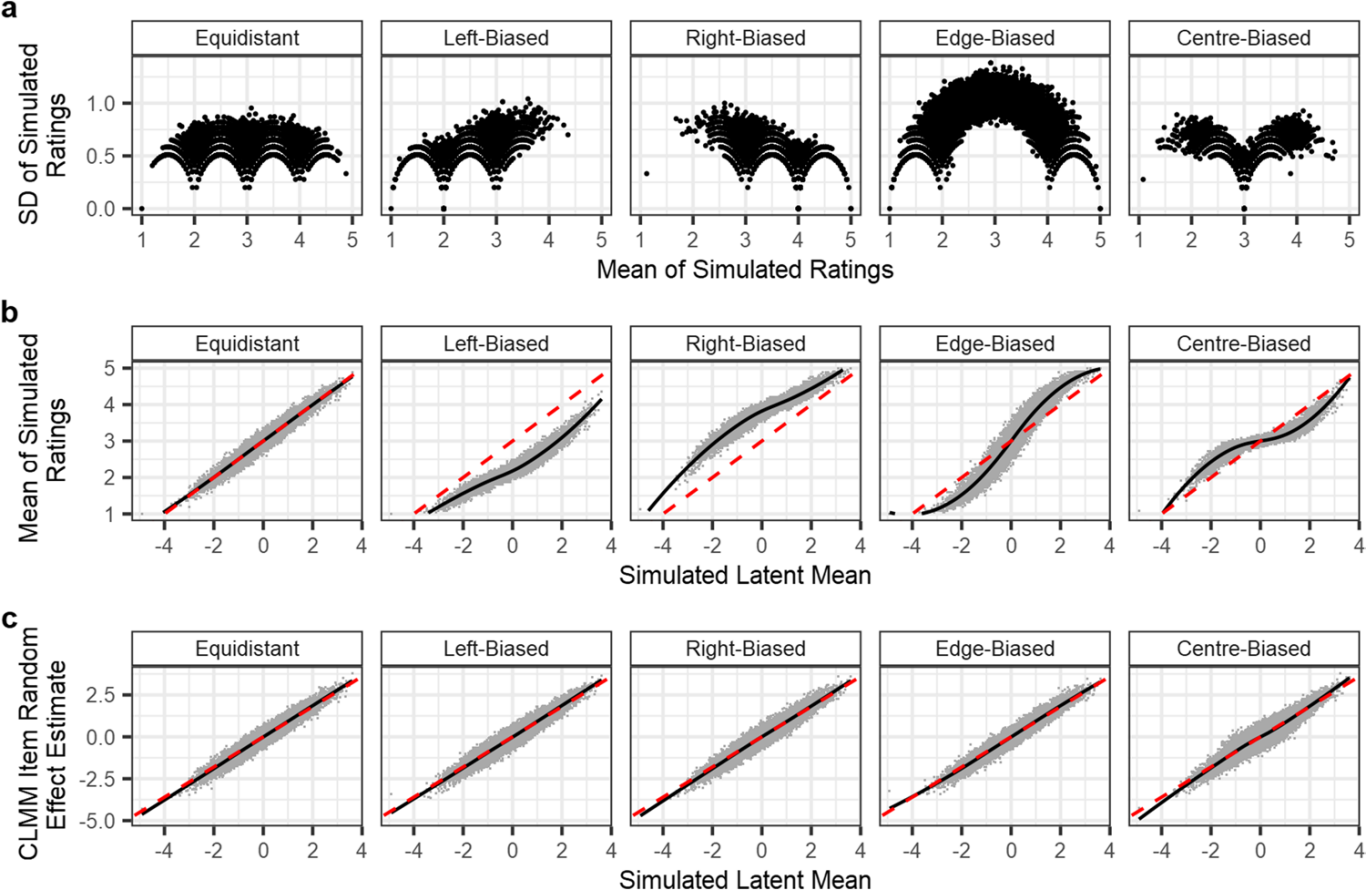
Results of Simulation 1: CLMMs recover items’ latent distribution random effects from the five example response patterns. The panels show: **a** the relationship between means and SDs of ratings, **b** the relationship between items’ simulated latent means and their mean ratings, and **c** the relationship between items’ simulated latent means and estimated random effects from the CLMM. The relationships in panels **b** and **c** shown with the *black lines* were estimated via locally estimated scatterplot smoothing (LOESS), with a span parameter of .75. The *dashed red lines* show an expected linear relationship for reference, identical across all response patterns. In all panels, results from all simulation iterations are concatenated. The results show that the relationship between items’ means and SD*s* of ratings differs markedly between simulated response patterns, even though the simulated values in the latent distribution were identical. While averaging over ordinal responses works well when the responses are generated from equidistant thresholds, any other response pattern leads to nonlinear inaccuracies in the values. CLMMs, meanwhile, account for any pattern of thresholds and more accurately recover the items’ distributions in the latent variable

Figure 5 depicts the results of Simulation 1. This simulation demonstrates that distinct patterns in the relationship between ratings’ means and SDs can arise from the response patterns alone, even when the underlying latent distribution is identical. The results further show that while the means of ratings are heavily influenced by nonlinearities in response patterns, estimates of item random effects from the CLMMs are more robust to differences between response patterns. We note, however, that the distortions that result from using the raw mean may be less problematic if researchers are only interested in rank order (see Supplementary Materials 1). Nevertheless, whenever researchers are interested in the relative distances between items, CLMMs provide estimates which are far less distorted by overall response patterns.

### Simulation 2: CLMMs with item and participant random effects

In the typical design of a rating norming study, the 25 observations simulated in the previous simulation would have come from different participants, who are likely to systematically differ in how they judge any given item. Because each participant would additionally rate a subset of the total set of items in the study, variability between participants can be calculated as an additional random effect. As a demonstration of this, we re-ran the previous simulation with the additional inclusion of participant random effects. As in the previous simulation, 25 observations were generated for each item, but each observation for an item was generated by 25 different simulated participants, where each participant rated 25 items in total. Participants were allocated to items pseudo-randomly, such that they rated each item only once. This meant there were a total of 100 participants in each iteration. The latent distribution values were thus simulated from a normal distribution with mean 0 and SD 1, with both item and participant random effects also drawn from normal distributions with mean 0 and SD 1. As a result, latent distribution value *L*_*ij*_ for the *i*th item and *j*th participant, was simulated as:

$${\displaystyle \begin{array}{c}{L}_{ij}={\mu}_i+{\mu}_j+{e}_{ij}\\ {}{\mu}_i\sim N\left(0,1\right)\\ {}\begin{array}{c}{\mu}_j\sim N\left(0,1\right)\\ {}{e}_{ij}\sim N\left(0,1\right)\end{array}\end{array}}$$

As before, ratings were simulated by recoding regions of the latent distribution using the five response patterns shown in Fig. 4. The CLMMs were again fit using the *ordinal* package with a probit-link function, specified to either (i) omit or (ii) include participant random effects in the formula, written in the package’s syntax as, respectively:

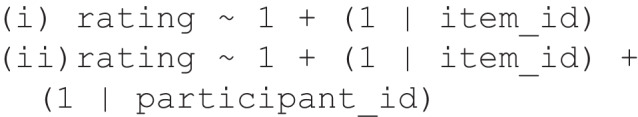



**Fig. 6:**
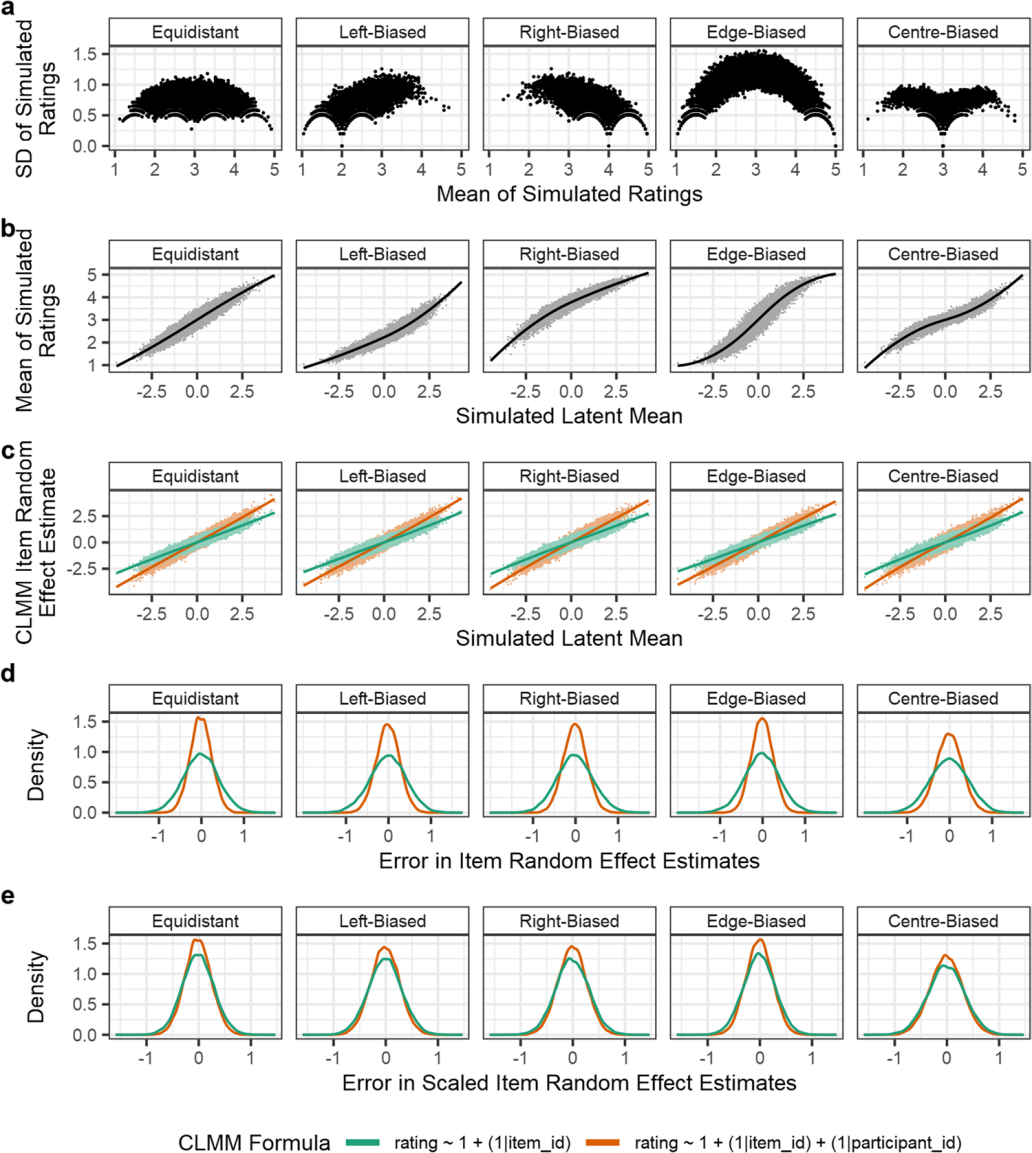
Results of Simulation 2: CLMMs recover items’ latent distribution random effects when per-participant random intercepts are also simulated. Panels **a** and **b** show the same information about items as the respective panels in Fig. 5, but for data which additionally simulated participant random intercepts. The additional variability from participant random effects has led to smoother patterns in panel **a**. Panel **c** shows the relationship between simulated latent distribution values and item random effect estimates, from the CLMM estimating item random intercepts only (*green*), and from the CLMM estimating both item and participant random intercepts (*orange*). As in Fig. 5, each *line* represents a LOESS estimate (span parameter of .75) of the relationship. Panel **d** shows the density of the error in the items’ random effect estimates (i.e., *error* = *simulated value* − *estimated value*) from both types of CLMM estimated. Here, observations from all 100 simulation iterations are concatenated. The distribution of the errors shows that including participant random effects improves the accuracy of estimates for item random effects. In panel **e**, this same difference is again presented, though the random effect estimates have been scaled by standard deviation to account for the differences in estimated magnitude shown in panel **c**

Figure 6 shows the Simulation 2 results, demonstrating that the estimation of participant random effects allows the CLMMs to recover the simulated item random effects more accurately.

In examining whether the estimation of participant random effects improves the accuracy of item random effect estimates, we first calculated the difference between each item’s simulated item random effect, and that estimated by the two models. As panel **d** of Fig. 6 shows, including both participant and item random effects in the fitted model improved the accuracy of the estimates compared to considering only item random effects. However, panel **c** shows that the magnitude of item random effects was underestimated when participant random effects were not calculated, which we considered could be the cause of the difference in accuracy of estimates between the two models. If the improvement in accuracy is due to a difference in magnitude alone, the improvement may not be meaningful or useful for rating norming studies. This is because it is the ordinal relationships and relative sizes of differences between items which are most informative. To examine whether the improvement in accuracy was solely the result of this difference in the magnitude of estimated item random effects, we calculated the error in item random effect estimates when model estimates are normalised by their respective SDs, thereby standardising the magnitude of the random effect estimates from each model. These results are presented in panel **e** of Fig. 6, showing that while most of the improvement in accuracy with participant random effects can be attributed to differences in the magnitude of effects, there may be some gain in accuracy when participant random effects are additionally accounted for.

**Fig. 7:**
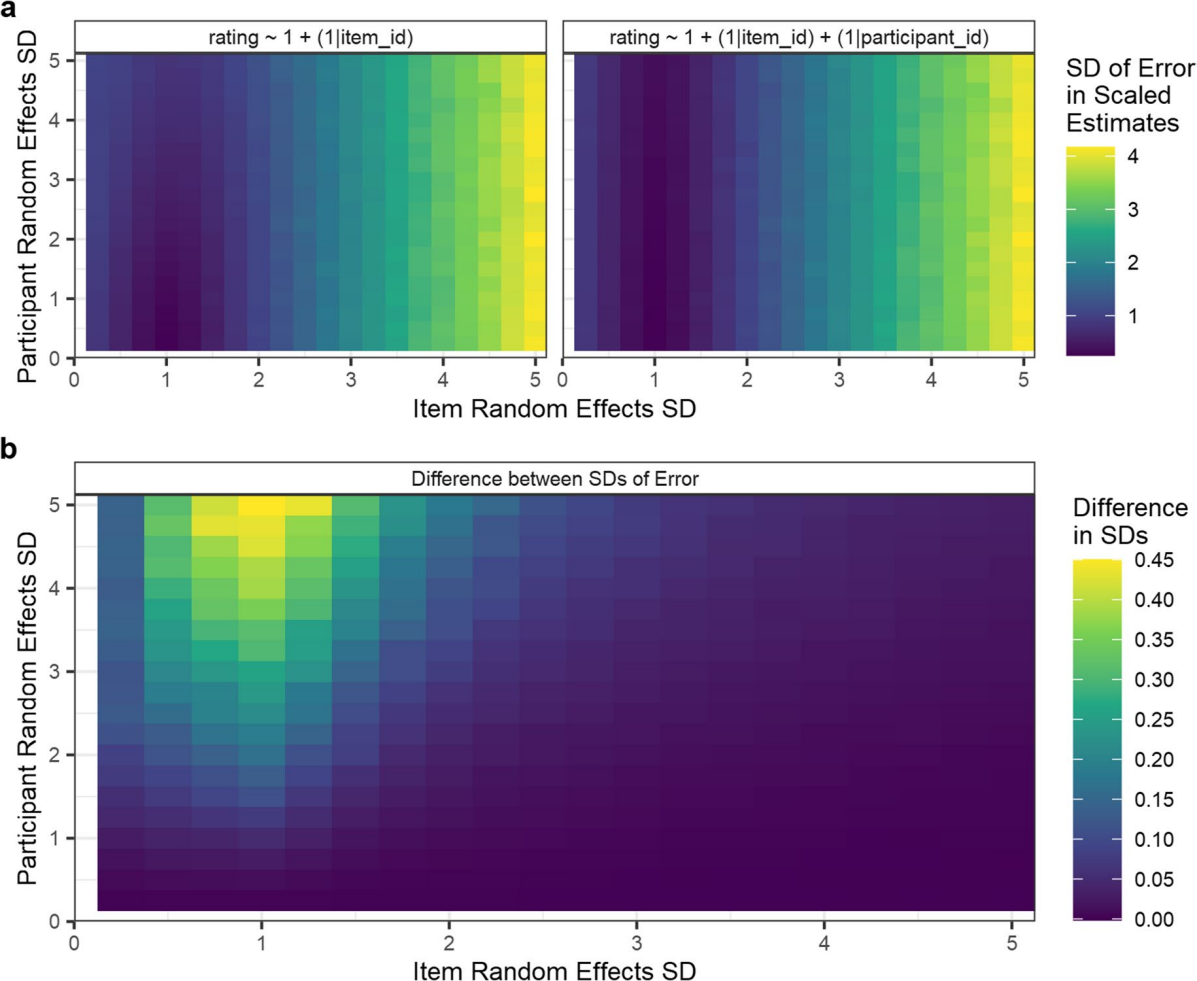
Results of Simulation 2b: effect of varying the magnitude of item (*x*-axis) and participant (*y*-axis) random effects on estimation accuracy of item random effects. Panel **a** shows the estimated SDs of errors of item random effects, where estimates are scaled (as in Fig. 6e) to account for differences in magnitude. Estimates in panel **a** are shown separately for a model estimating only item random effects (*left*), and a model estimating both item and participant random effects (*right*). Panel **b** shows the difference between the estimates from the two models, calculated as item estimates from the less complex model (estimating only item random effects) minus those of the more complex model (estimating both item and participant random effects). Values in panel **b** therefore index the reduction in error that results from accounting for participant random effects (e.g., a value of .3 reflects a reduction of .3 SDs in the magnitude of errors)

The degree to which item random effect estimates increase in accuracy when participant random effect estimates are included will depend on features of the data. One important consideration is the magnitude of variances of the random effect distributions relative to one another, and to the latent distribution. This is because greater variance in the participant random effects distribution will increase the degree to which estimates are distorted by the biases of individual participants. To demonstrate this, we ran additional iterations in our Simulation 2b (see Fig. 7), varying the SDs of the participant and item random effect distributions from which the random effects are simulated. For simplicity, and because there would be similar results for each response pattern, we simulated Likert responses from the edge-biased response pattern only. SDs of item and participant random effect distributions were varied between .25 and 5, in steps of .25. All other features of the data were simulated as specified above. We ran 50 iterations for each combination of item and participant random effects and calculated the SDs of the error in scaled item random effect estimates (Fig. 7a). We could then calculate the difference between these estimates to estimate the effect of including participant random effects on the accuracy of item random effects (Fig. 7b). This analysis revealed that estimating participant random effects in the CLMM random effect structure can reduce error in the estimates of item random effects. Specifically, the results suggest that when participants are more variable than items, estimating participant random effects increases the accuracy of item random effects estimates. When items are more variable than participants, the results suggest that while there is no gain in accuracy, there is also no loss of accuracy.

An alternative approach to accounting for participant variability when calculating item norms may be to first *z*-score responses within participants, before calculating per-item averages. We consider such an approach in our Supplementary Materials 2. To summarise this evaluation, we conclude that such an approach is well considered as a simple approach which will account for per-participant differences in central tendency, but that in itself it fails to account for the ordinal nature of a Likert scale, and will accordingly result in a similar distortion of estimates to that observed for raw averages. We further argue that CLMMs provide additional advantages, such as estimating item and participant variability concurrently, rather than accounting for these sources in separate steps, as the *z*-scoring approach does.

In sum, we have shown that estimating both item and participant random effects can improve the accuracy of item random effect estimates from a CLMM applied to data with a design comparable to that of a typical rating norming study. Fitting CLMMs which estimate item as well as participant random effects is unlikely to *reduce* accuracy of estimates and will provide a gain in accuracy which is dependent on the relative variability of items and participants. As a result, we argue that modelling both sources of variability simultaneously is both useful and appropriate when the goal is to accurately norm items on the basis of Likert ratings.

### Simulation 3: CLMMs estimating latent variance

All CLMMs shown thus far estimate changes in the mean of a latent normal distribution, assuming homogeneity of variance. The latent distribution’s spread may also differ meaningfully between items, however. As an example, consider polysemous words (words with multiple senses): for example, the word *lie* may be used in the sense of a bodily position, or in the sense of spreading falsehoods. As a result, one may expect ratings on semantic dimensions to show greater variance for such ambiguous words. However, if the words are presented with a disambiguating context (e.g., *lie (position)* and *lie (untruth)*), one may expect not only an associated shift in the average of Likert ratings (Scott et al., 2019), but also in the variance of ratings. Like means, however, SDs of Likert ratings incorrectly assume continuity in an ordinal scale, and this accordingly causes response patterns to distort estimates (see panel **a** of Figs. 5 and 6). As with the mean, an estimate of the SD of the latent distribution may therefore be used to disentangle such meaningful differences from the ordinal response pattern.

Although packages like *ordinal* generally assume homogeneity of variance, differences in multiple parameters of a distribution function can be modelled concurrently with the *brms* package (Bürkner, 2018) for R. When estimating changes in all parameters which specify a distribution, such an approach can be considered an example of distributional modelling. Extending this method to CLMs and CLMMs can allow researchers to estimate differences in a latent distribution’s mean and variance concurrently. Here, the latent distribution’s mean is estimated on an identity scale via one linear formula, while a second linear formula allows differences in the latent distribution’s variance to be estimated as changes in a *discrimination* parameter (Bürkner, 2020). This parameter is specified as the inverse of the latent distribution’s SD (i.e., 1/SD), and is modelled on a log scale by default (Bürkner & Vuorre, 2019). In the previous simulations, we have shown that random effect estimates of an item’s mean in a latent distribution can be used as a measure of its central tendency in the rated dimension, akin to means of Likert ratings but disentangled from overall response biases. In a similar manner, we argue that random effect estimates of the latent distribution’s *variance* can be used as a measure of an item’s spread in the rated dimension, akin to the SD of Likert ratings, but again, disentangled from response patterns.

To demonstrate that a distributional CLMM can accurately estimate items’ latent means and SDs across different response patterns, we simulated data with participant and item random effects for both the latent distribution’s mean and SD. The numbers of participants and items, and the numbers of observations per participant or item, were identical to those used in Simulation 2. The latent distribution value associated with each trial, however, was simulated as follows:

$${\displaystyle \begin{array}{c}{L}_{ij}\sim N\left({\mu}_{ij},{\sigma}_{ij}\right)\\ {}{\mu}_{ij}={\mu}_i+{\mu}_j\\ {}\begin{array}{c}{\mu}_i\sim N\left(0,1\right)\\ {}{\mu}_j\sim N\left(0,1\right)\\ {}\begin{array}{c}{\sigma}_{ij}=\frac{1}{e^{disc_i+{disc}_j}}\\ {}{disc}_i\sim N\left(0,0.5\right)\\ {}{disc}_j\sim N\left(0,0.5\right)\end{array}\end{array}\end{array}}$$

Here, latent values (*L*_*ij*_) are drawn from a normal distribution with mean *μ*_*ij*_ and SD *σ*_*ij*_. Latent means (*μ*_*ij*_) are calculated as the sum of item (*μ*_*i*_) and participant (*μ*_*j*_) random effects, which are both drawn from normal distributions of mean 0 and SD 1. Latent SDs (*σ*_*ij*_) are calculated as the inverse of the exponent of the sum of item (*disc*_*i*_) and participant (*disc*_*j*_) random effects for a discrimination parameter, which are drawn from normal distributions of mean 0 and SD .5.

**Fig. 8:**
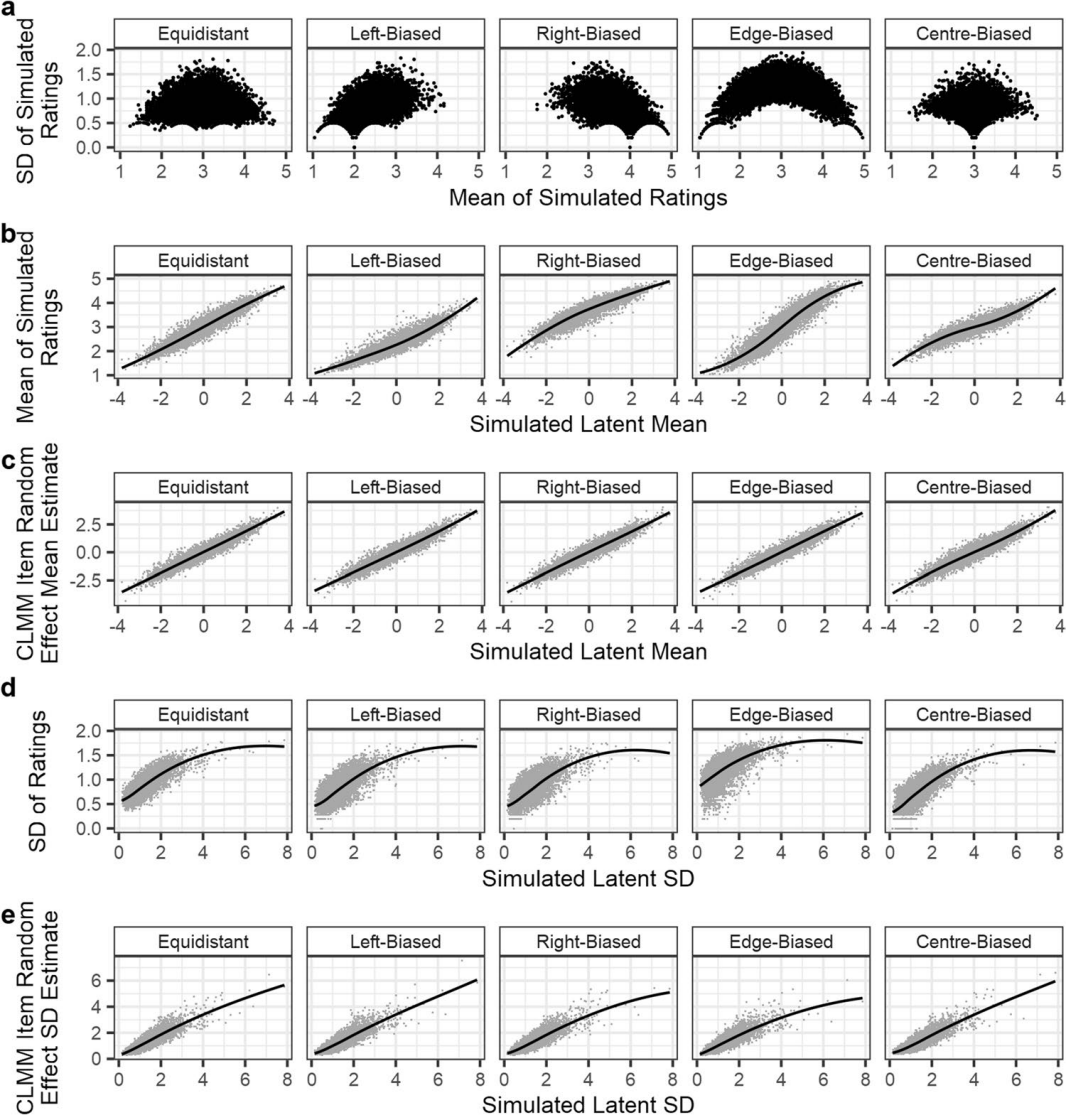
Results of Simulation 3: efficacy of distributional unequal variance CLMMs for calculating the SD of latent variables’ variance from Likert response data. Panels **a**, **b**, and **c** show that the findings from the previous simulation also apply to models which calculate differences in both the mean and variance of the latent distribution. Panel **d** shows the relationship between items’ simulated latent variable SD, and the SD of Likert ratings, while panel **e** shows that differences in the latent distribution’s SD are more accurately retrieved by random effects in the *disc* parameter. *Lines* tracking relationships in panels **b**–**e** are estimated via LOESS (span parameter = .75)

In total, 100 datasets were simulated, and, as in the previous simulations, the latent distribution was recoded to values on the Likert scale using the five different response patterns. For each of the five response patterns in each of the 100 iterations, a probit-link Bayesian distributional CLMM was fit with *brms*, with 3 Markov chains consisting of 6000 iterations each (split equally between warmup and sampling). For all CLMMs, the *adapt_delta* parameter was set to .8, and the *max_treedepth* parameter was set to 10. In *brms* syntax, the model formula was specified as follows:



The results of Simulation 3 are presented in Fig. 8. As in the previous simulations, averages of simulated Likert responses were distorted by nonlinearities in response patterns (panel **b**), whereas CLMM random effects scaled linearly, across all response patterns, as a function of simulated differences in the latent variable (panel **c**). Similarly, SDs of simulated Likert responses less accurately represented the simulated latent variable variance (panel **d**) than SDs calculated from random effects for the *disc* parameter (panel **e**). Notably, as the simulated latent variable SDs increase, the degree to which this is underestimated by SDs of Likert ratings increases, to the extent that items with a simulated SD of 8 are only estimated as having an SD of between 1.5 and 2. This is a consequence of the Likert scale’s finite bounds.

The results of Simulation 3 show that unequal variances in the latent distribution can be retrieved by a distributional CLMM.

**Fig. 9:**
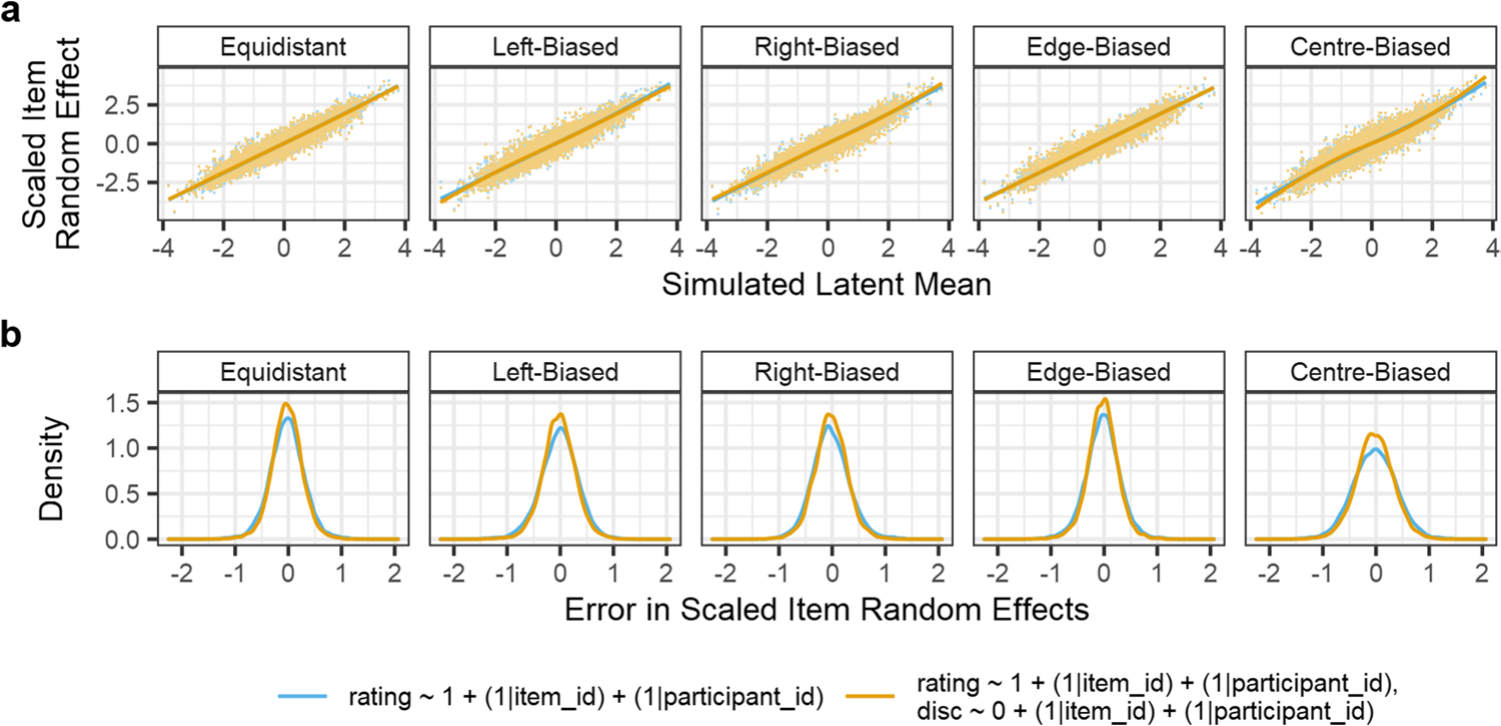
Comparison between a CLMM assuming equal variance across observations (*blue*), and a CLMM estimating differences in the variance of the latent distribution (*yellow*). Panel **a** shows that a similar pattern exists for both models between simulated latent means, and those estimated by the models’ random effects (scaled for comparability between the models). The estimates are so similar that results for the equal variances model are largely overlaid by results from the distributional model. Panel **b** shows the density of the differences between simulated latent mean values and scaled estimates from CLMM random effects. While estimating items’ differences in latent variance may provide a small gain in accuracy for the estimated means, reflected by the slightly heavier tails in the density plots, this improvement is minimal for data simulated here

However, a model assuming *equal* variances across observations can still accurately retrieve differences in the central tendency of the latent distribution, when variances differ systematically between participants and items. To demonstrate this, for each of the 100 datasets in this simulation, we fit an additional model assuming equal variance across observations. For comparability, this model was specified and fit in a manner identical to that of the distributional CLMM (i.e., using *brms* with identical sampling settings). The sole difference between the model specifications was that the formula for the equal-variance model only estimated differences in the latent distribution mean, assuming homogenous latent variance. In *brms* syntax, this was simply written as follows:



We could then compare the accuracy of estimation of each item’s latent distribution mean, from each iteration of the simulation. The results of this analysis are summarised in Fig. 9, showing that accuracy in the estimation of item random effects is very similar for both equal and unequal variance models. As a result, assuming equal variance, when variances across items and participants are in fact unequal, may not be overly problematic, provided the researcher is not interested in the differences in variance of the latent distribution. However, the extent to which accounting for unequal variance may improve accuracy of estimates may be related to the magnitude of differences in latent variance, relative to the magnitude of differences in latent mean. Consequently, researchers should carefully consider whether they expect meaningful differences in the variance of the latent distribution.

### Simulation 4: Robustness of the normal assumption

The previous simulations all considered a normally distributed latent variable. This choice was motivated by normality of the latent distribution being a central assumption of CL(M)Ms fit with a probit-link function. Other link functions similarly assume other distributions; for instance, the logit-link function assumes the latent variable takes a logistic distribution. Relatedly, CLMMs assume that item and participant random effects are drawn from normal distributions centred on zero. One can imagine scenarios, however, in which a model’s distributional assumptions, for either the latent variable or random effects, are inconsistent with reality. For example, item random effects may be bimodally distributed if there are two distinct categories in the data, such as has been argued to be the case for judgements of concreteness (Pollock, 2018). To demonstrate that the use of CLMMs for norming items is relatively robust to violations of the models’ distributional assumptions, we ran two simulations fitting probit-link CLMMs via the *ordinal* R package (assuming equal variances) to data where, respectively, the latent variable (Simulation 4a) or the item random effects (Simulation 4b) are drawn from non-normal distributions. In all simulations, for simplicity of the results, we simulated only the edge-biased response pattern.

**Fig. 10:**
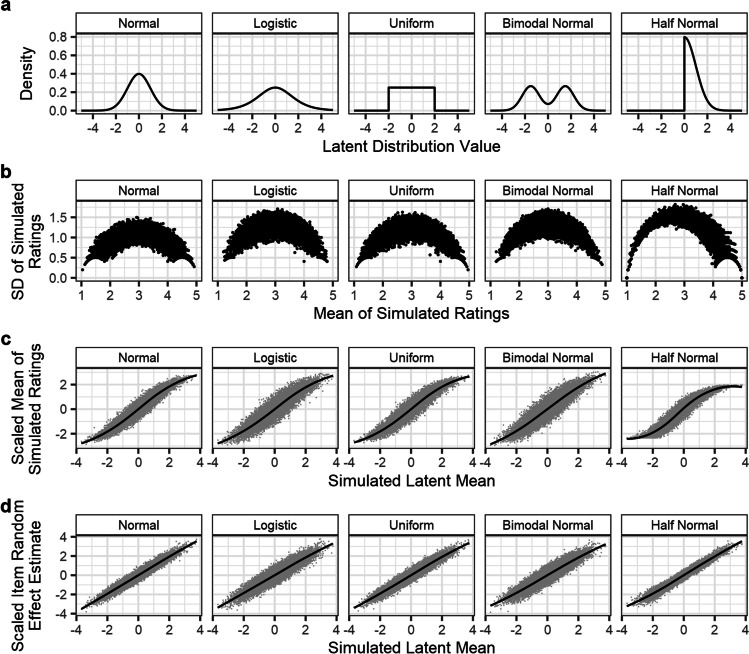
Results of Simulation 4a: varying the shape of the latent distribution. Across all non-normal distributions (panel **a**), a similar heterogenous pattern in the mean–SD relationship in Likert ratings was observed, though it is notably asymmetrical in the case of the half-normal distribution (panel **b**). For all distributions, estimates of items’ simulated latent variable values were distorted by the edge-biased response pattern when estimated via the mean of simulated Likert ratings (panel **c**). In contrast, random effects estimates from the CLMM more accurately retrieved the simulated latent variable values, with similar accuracy across the distribution (panel **d**)

In these final two simulations, the data was generated equivalently to that described in Simulation 2, except that either the latent distribution, or the item random effects, were drawn from one of five possible distributions (Fig. 10a). The first of the five distributions was a normal distribution with mean of zero and an SD of one (i.e., identical to the *N*(0, 1) used in Simulation 2). This was included such that results for all other distributions could be directly compared to data which conformed to the model’s assumptions. The four non-normal distributions were as follows: a logistic distribution (*μ* = 0, *s* = 1); a uniform distribution (min = – 2, max = 2); a bimodal distribution composed of two normal distributions (respectively, *μ* = – 1.5, *σ* = .75, and *μ* = 1.5, *σ* = .75); and a half-normal distribution (*μ* = 0, *σ* = 1). Each of the two simulations was run for 250 iterations. In both simulations, the distributions of either the latent variable or item random effects was altered while all other parameters and results were held constant.

In the first of the two simulations (Simulation 4a), we varied the distribution that the latent variable is drawn from. Item and participant random effects distributions, meanwhile, were drawn from the normal distributions specified in Simulation 2. Figure 10 presents the results of this simulation: using the same (probit-) link function to model data, where the latent variable values are in fact drawn from the five distributions described above, may affect the accuracy of estimation of item random effects, but in all cases provides item norms which are more accurate, and scale more linearly, than calculation of mean Likert responses.

**Fig. 11:**
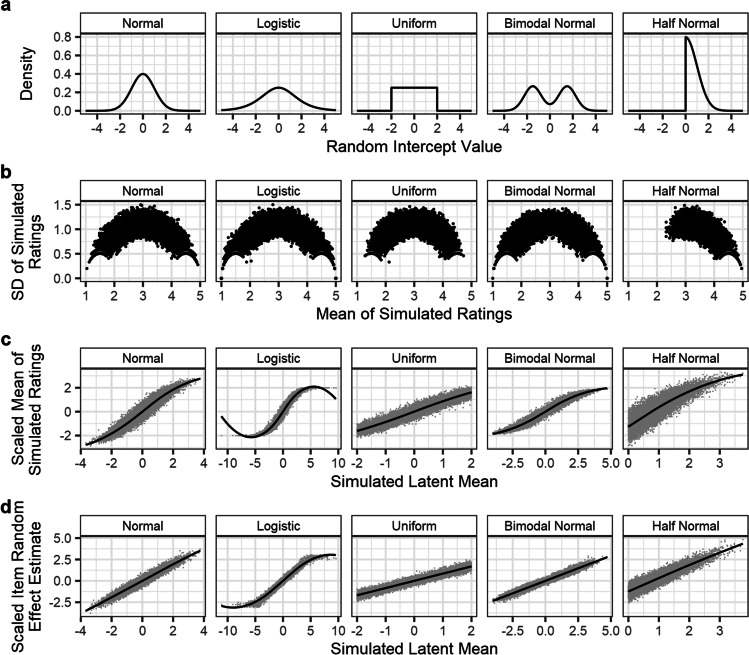
Results of Simulation 4b: varying the distribution of the item random effects. Across all non-normal distributions (panel **a**), the mean–SD relationship for Likert ratings (panel **b**) showed greatest inconsistency at the midpoints of the Likert scale, although this reflects any asymmetries in the random effect distribution, as is shown for the half-normal distribution. For all non-normal random effect distributions simulated, estimates from averages of Likert ratings (panel **c**) are distorted by the response pattern, and are less accurate than random effect estimates from CLMMs (panel **d**), which scale more linearly with simulated values. We note that the unusual pattern in panel **c** for random effects drawn from a logistic distribution does not reflect the pattern of observations well, but is an artefact of the LOESS method of estimation

In the second of the two simulations (Simulation 4b), we varied the distribution that the simulated item random effects were drawn from. The latent distribution itself, and the distribution of participant random effects, were simulated as the normal distributions described in Simulation 2. Figure 11 presents the results of this simulation, showing that, even for very non-normal random effects distributions, the CLMMs still estimate the item random effects more accurately than would a traditional average of Likert ratings.

## Application to real data

To demonstrate the viability of CLMMs for norming items, we applied them to a real dataset collected from a norming study. We modelled character similarity judgements collected by Simpson et al. (2013), which show a similar mean–SD relationship to that identified by Pollock (2018). We also demonstrate that CLMMs show patterns of results like those in our simulations, and that, unlike traditional summary statistics of Likert responses, they can disentangle meaningful differences among items from participants’ overall response patterns.

Simpson et al. (2013) collected character similarity judgements for 2704 pairs of characters, from 677 participants, on a seven-point Likert scale ranging from not at all similar (*1*) to very similar (*7*). Each pair of characters comprised either two lower-case or two upper-case characters. The trial-level data, shared via personal correspondence, consisted of 81,199 total trials, with between 108 and 120 trials per participant, and between 29 and 31 ratings per item. We note that unlike the original analysis, we did not exclude responses based on a ± 2 SD cut-off, but only excluded missing (i.e., blank) or meaningless (e.g., less than *1* or more than *7*) responses.

We fit a Bayesian distributional CLMM to the trial-level data, estimating item and participant random effects. The model was fit with *brms*, using a cumulative probit-link function, and with six Markov chains of 6000 iterations each (split equally into warmup and sampling). The *adapt_delta* parameter was set to .95, and the *max_treedepth* was set to 10. To reduce the size of the model for feasibility of storage, the thin argument was set to 2, meaning that only one half of the posterior samples (i.e., 1500 per chain) were saved. In *brms* syntax, the model formula was as follows:

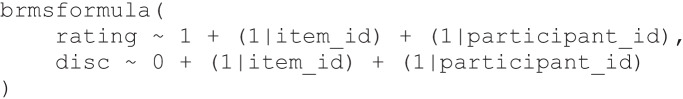



**Fig. 12:**
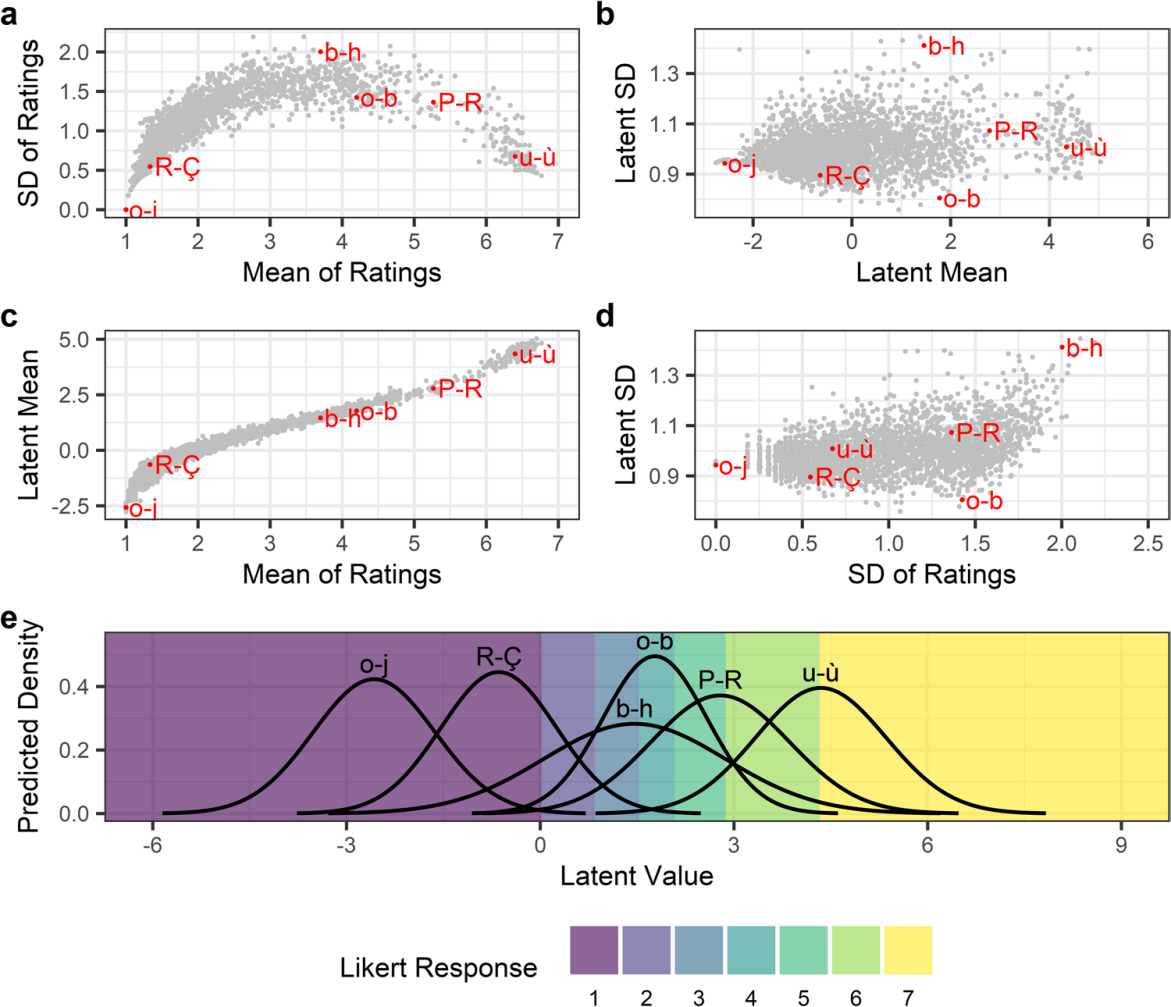
Modelling of character similarity ratings from Simpson et al. (2013): Item random effect results of the Bayesian distributional CLMM. Six pairs of Arial characters are highlighted as examples, ranging from *o-j* at a low level of similarity, to *u-ù* at a high level of similarity. Panels depict: **a** the mean–SD relationship in items’ ratings; **b** the mean-SD relationship in the latent distribution; **c** the relationship between each item’s mean rating and its latent distribution mean as estimated by the CLMM; **d** the relationship between ratings’ SDs and the estimated latent SDs; and **e** the predicted densities of the latent distributions for the six example items, calculated from their random effect estimates for the *mu* and *disc* parameters. Coloured regions indicate the mapping from latent values to Likert responses, where the boundaries between coloured regions reflect the estimated locations of the latent thresholds

Table 1 presents the estimates and credible intervals for parameters estimated by the model. A summary of the per-item results is presented in Fig. 12, showing that the problems we identified with reporting means and SDs for ordinal Likert responses, namely the distortion of estimates by response patterns, did affect the data. Notably, the patterns of mean–SD relationships (Fig. 12a), and the estimated locations of the thresholds (Fig. 12e), are similar to those we observed in simulations of edge-biased responses (Fig. 5). Similarly, given that latent means provide a measure less biased by response patterns, the relationship between means of Likert ratings and latent means (Fig. 12c) suggests that the use of averaged ordinal responses has distorted the results of the norming study. Furthermore, this nonlinearity in the study’s response pattern is accounted for by the CLMM, with the distinct inverted U pattern (Fig. 12a) disappearing for means and SDs in the latent distribution (Fig. 12b). However, some differences between items’ SDs of Likert responses is preserved in latent SDs (e.g., between items *o-b* and *b-h*), suggesting that SDs of ratings are influenced by both response patterns and variability of responses for different items. This is reflected in the scatter plot showing the relationship between SDs of ratings and latent SDs (Fig. 12d), which suggests only a noisy relationship, due to the influence of response patterns on SDs of Likert ratings. In summary, by estimating differences in the mean and SD of the latent distribution, we were able to estimate analogues to the traditional mean and SD of Likert responses, but which are disentangled from the raters’ response biases. These estimates of latent means and SDs can be used to calculate a latent distribution for any presented item (Fig. 12e). When combined with the threshold estimates, it is possible to probabilistically predict Likert responses for any item. For instance, the character pair *u-ù* would be expected to elicit a Likert response of *7* around 50% of the time.

**Table 1:** Modelling of character similarity ratings from Simpson et al. (2013): estimates (medians of posterior distributions) and 89% credible intervals (89% highest density intervals) for the key parameters estimated by the Bayesian distributional CLMM. The first six parameters reflect the estimated locations of the six thresholds in the latent distribution (e.g., *Threshold 3|4* reflects the latent location of the threshold between Likert responses for *3* and *4*). The last four parameters reflect the standard deviation of the random effects distributions for the cumulative link’s *mu*, and *disc* parameters, for items (*i*) and participants (*j*). The symbols and *i* and *j* subscripts are used for consistency with the simulations. These estimates revealed that magnitude of differences is larger in the *mu* parameter than in the *disc* parameter, and interestingly, that the variability in *mu* is larger for items than for participants, while the variability in *disc* is larger for participants than for items

Parameter	Estimate	89% credible interval
Threshold 1|2	.02	[– .04, .08]
Threshold 2|3	.84	[.77, .91]
Threshold 3|4	1.53	[1.46, 1.60]
Threshold 4|5	2.08	[2.00, 2.15]
Threshold 5|6	2.87	[2.79, 2.96]
Threshold 6|7	4.33	[4.22, 4.44]
SD of *μ*_*i*_	1.47	[1.43, 1.51]
SD of *μ*_*j*_	.71	[.67, .75]
SD of *disc*_*i*_	.16	[.15, .17]
SD of *disc*_*j*_	.27	[.26, .29]

**Fig. 13:**
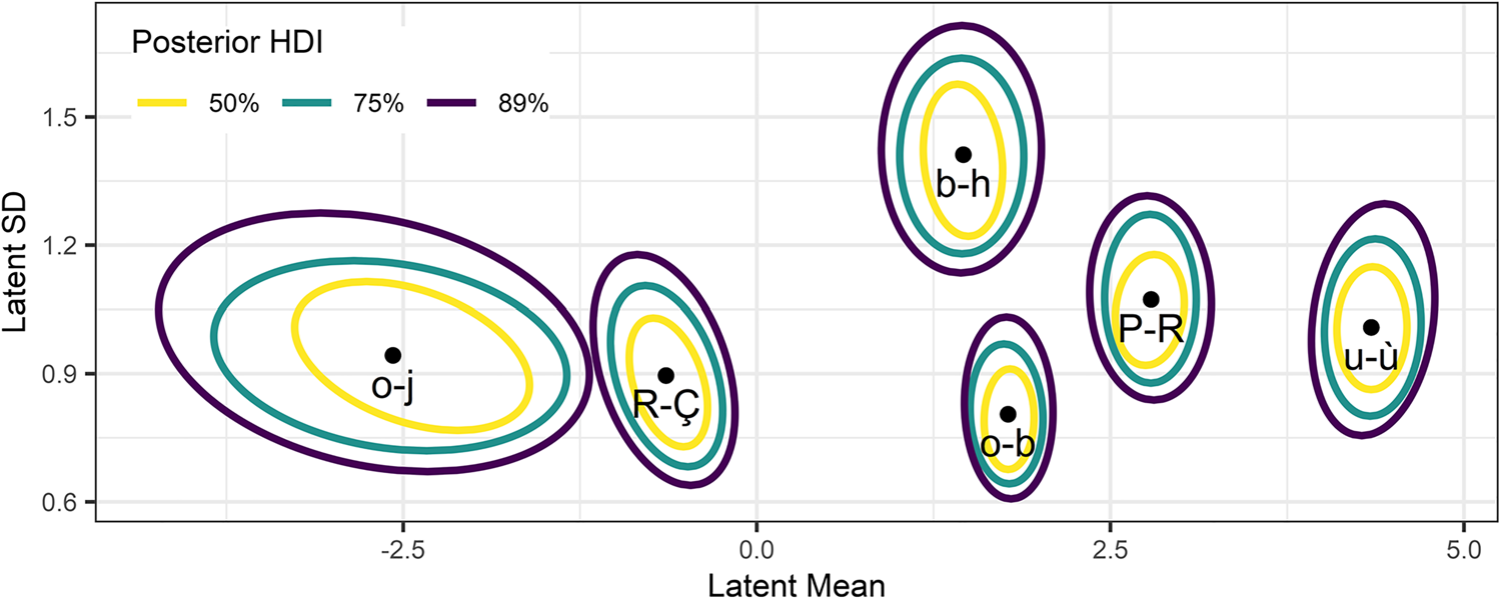
Uncertainty in the estimates of example pairs of characters’ means and standard deviations in the latent distribution. *Ellipsoids* present the 50, 75, and 89% highest density intervals (HDIs) in the posterior samples, while *points* show the median estimates for each pair of characters

The use of Bayesian estimation in the distributional model can also allow researchers to examine uncertainty in the random effects of each item. To demonstrate this, we calculated a series of two-dimensional highest density intervals for the latent distribution’s mean and SDs, for each of the example items highlighted in Fig. 12. These highest density intervals are presented in Fig. 13 and demonstrate the degree of uncertainty in the posterior estimates of the Bayesian distributional model. For instance, Fig. 13 shows that of the six example items, we are most certain about the latent mean and SD values for the *o-b* pair. In contrast, we are very uncertain about the latent mean and SD associated with the *o-j* pair. This is in part likely to reflect that all responses were *1* for this pair, such that a floor effect makes it difficult to estimate the latent distribution (i.e., there are many normal distributions which could plausibly result in the observed number of participants consistently responding with the lowest value in the Likert scale).

The Bayesian distributional CLMM presented above modelled differences in both latent means and latent variances. Following the results of Simulation 3, we were interested in examining how our estimates would change if the CLMM assumed equal variance across observations. To additionally show the similarity in the results between Bayesian MCMC and frequentist maximum likelihood models, we fit an equal-variance model using the *ordinal* package. The model was fit using the same link function as the Bayesian distributional CLMM (probit), with a random effects structure specified as follows:

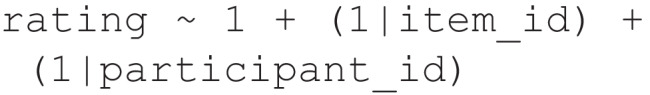



**Fig. 14:**
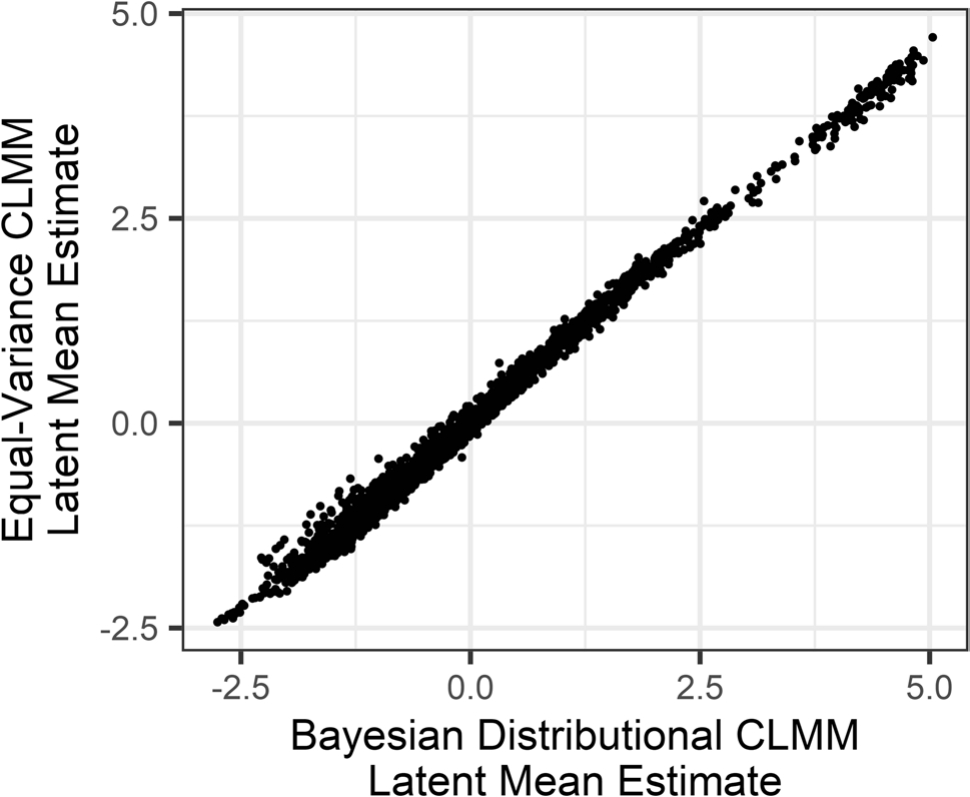
The latent mean estimates from the Bayesian distributional CLMM, fit with the *brms* package, correlate very highly with estimates from the model assuming equal variance, fit with the *ordinal* package

We could then compare the per-item latent mean estimates of the two models. In this way, we could see to what extent assuming equal variance (and fitting via maximum likelihood rather than Bayesian estimation) would affect our estimates if we were only interested in latent means. This revealed a Pearson’s correlation of *r* = .997 between the models’ estimates (Fig. 14). However, it is worth noting that there were considerable differences in the time it took to fit each of the two models – the Bayesian distributional model took several hours to fit on a typical modern computer, while the equal-variance model fit with maximum likelihood took only a couple of minutes. Nevertheless, the simpler, equal-variance model provided extremely similar estimates of per-item latent means. Had we only been interested in latent means, the simpler, equal-variance model would have been arguably sufficient for norming the similarity judgements, though that would mean forfeiting the rich posterior distributions afforded by the Bayesian approach.

**Fig. 15:**
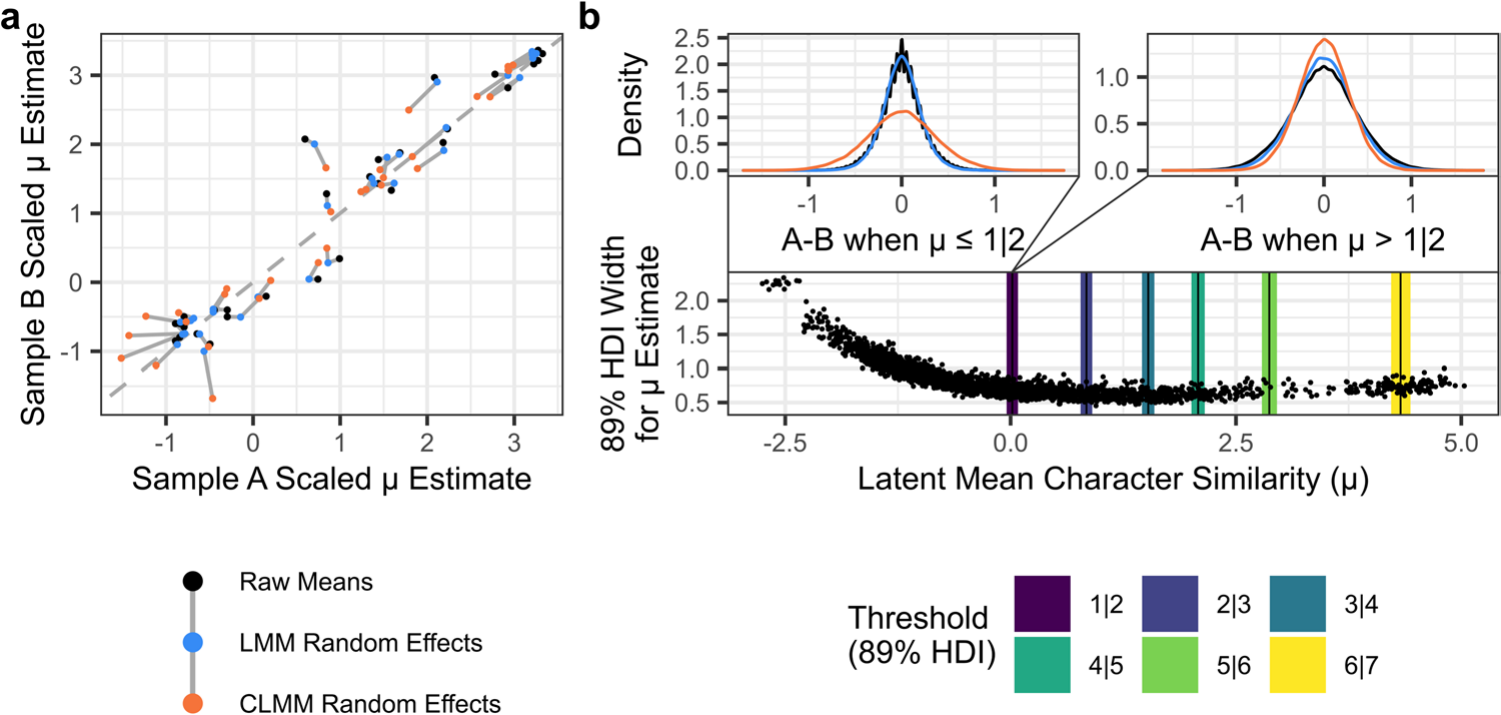
Norm consistency. Results of the analysis examining consistency in norms estimates for three approaches: raw means, LMM random effects, and CLMM random effects. Panel **a** depicts the test–retest consistency for 30 example items, from one test–retest iteration. Estimates for the same items, from the three different approaches, are joined by *grey lines*, while the *dashed diagonal line* depicts perfect consistency between the two samples for reference. The lower region of panel **b** shows how uncertainty in latent mean estimates (width of the 89% HDI) from the Bayesian distributional model varies across the latent scale. The *coloured bands* depict the 89% HDIs for the estimated threshold locations (e.g., 1|2 is the threshold between ratings of *1* and *2*). The upper region of panel **b** shows the distribution of differences between samples A and B, with observations combined from 100 iterations of the test–retest procedure. The differences are depicted separately for items which were either below (*left*) or above (*right*) the lowest threshold. The jagged appearance of the density plot of differences for raw means reflects that there is only a finite number of possible values the average rating can take without the additional discriminatory power provided by random effects

We were finally interested in characterising the variability of norms derived via the CLMM approach between separate samples. To this end, we used a method of cross-validation whereby the full Simpson et al. dataset is split randomly into two samples, with roughly equal numbers of ratings for each item, and roughly equal numbers of trials for all items per sample. Both samples could then be normed independently, so that consistency of estimates across samples could be examined (Fig. 15a). We compared the results from CLMM-derived estimates to both raw means, and estimates derived from the random effects structure of a linear mixed effects model (LMM). This latter comparison was employed to delineate the effects of shrinkage and of accounting for participant variability which result from the random effects structure (LMM vs. raw means) from the effect of treating the scale as ordinal rather than continuous (CLMM vs. LMM). The LMMs were fit via the *lme4* package for R (Bates et al., 2015), with a Gaussian identity link for comparability to the raw means. Both CLMMs and LMMs were fit with item and participant random intercepts. The process of splitting the data in two, and estimating norms for both samples using each of the three methods, was repeated 100 times. We could then examine the distribution of differences between the two samples of all iterations (Fig. 15b). We expected this distribution of differences to have less of a spread when results are more consistent, and to have greater spread when results are less consistent. We found that across most of the Likert scale, the CLMM-derived norms were more consistent between separate samples. The exception was at the lower end of the scale, where items were overwhelmingly responded to with a rating of *1* (i.e., “not at all similar”). Here, we found that raw means and estimates derived from LMMs were in fact *more* consistent than estimates derived from CLMMs. However, we argue that this consistency is in fact illusory, since raw means and LMMs fail to account for the finite bounds of the Likert scale, resulting in very small variance estimates due to floor and ceiling effects. In contrast, the CLMM approach provides greater discriminatory power, by estimating differences in a latent distribution which does not suffer from the same bounds. The CLMM-derived estimates are therefore necessarily more variable in the extremes of the scale, where non-ordinal approaches would typically suffer from floor or ceiling effects. In such cases, the estimates provided by CLMMs are more informative than estimates from approaches like raw means and LMMs. Indeed, in the Bayesian distributional model we found that for items affected by floor effects, uncertainty in the latent mean increases markedly with distance from the location of the lowest threshold (lower section of Fig. 15b). In such cases, the responses are too consistent to provide much statistical certainty, and estimates increasingly rely on other, less directly informative features in the data. For example, items’ latent means will be adjusted based on the random effects of participants who provided the items’ ratings (as in Simulation 3). This suggests another advantage of Bayesian modelling, in that it can allow researchers to describe the certainty of their estimated norms. However, we note that comparable measures could be calculated for non-Bayesian models via a Monte-Carlo reanalysis of the results. Moreover, the increased uncertainty observed at extreme values highlights the importance of considering the design of rating scales and wording of instructions provided to participants. By carefully wording the task’s instructions or anchoring responses with labels (Hollis & Westbury, 2018), researchers may be able to systematically shift participant’s responses away from a floor or ceiling effect, such that differences in the probabilities of the possible ratings allow items to be normed with greater certainty, consistency, and precision. Nevertheless, this analysis demonstrates that a CLMM approach to norming items is more robust to floor and ceiling effects than non-ordinal alternatives.

All code, and the fitted Bayesian model, for this reanalysis of the data from Simpson et al. (2013) is available in the OSF project associated with this article, at https://osf.io/ntvmf/. This project additionally contains RMarkdown documents showing and explaining minimal examples of how to use CLMMs to norm items. Researchers unfamiliar with R, who wish to compare raw to CLMM-derived estimates, may also be interested in a web app created to promote norming items via CLMMs: https://github.com/JackEdTaylor/shinynorms. This app provides functionality to fit equal-variance CLMMs via the *ordinal* package, with the item and participant random effects downloadable alongside traditional summary statistics of means and SDs.

## Discussion

Norming studies which use ordinal scales constitute a vital resource for research and are applied to a wide range of scientific applications. It is therefore important that the reported norms accurately reflect the inter-item relations on the dimension of interest. Informed by the simulations reported here, we argue that to this end, norming studies should report estimates which appropriately account for nonlinearities in the ordinal norming scale, rather than inaccurately assuming that the ordinal scale is linear. Specifically, we have shown that, when using an ordinal Likert scale to norm items, traditional methods such as raw means and SDs can lead to systematically distorted item comparisons. On the other hand, properly accounting for the ordinal nature of the judgements via CLMMs provides estimates which are far less affected by participants’ response mappings. While this problem is well understood in studies which aim to estimate fixed effects of experimental manipulations (Liddell & Kruschke, 2018), the problem has been less widely discussed in relation to norming studies. Our contribution has been to show that by extracting item random effects, CLMMs can be used to more accurately norm items.

Our chief recommendation is that items are normed via the random effects structure of hierarchical ordinal models like CLMMs. In this way, researchers will be able to estimate norms which are appropriately disentangled from the nonlinearities in response patterns. However, we argue that while random effects estimates extracted from CLMMs will provide more accurate estimates for norming studies, such studies should report them in *addition* to more traditional measures like Likert means and SDs, rather than *instead* of them. This will be important for ensuring that results are still comparable with existing datasets which only report means and SDs, and for users of the data to examine the differences between estimates from ordinal models versus traditional summary statistics.

We also encourage researchers to share trial-level data from norming studies, in addition to per-item summaries. In this way, other researchers will be able to model data in an exploratory manner, or with models more appropriate to their research questions (e.g., including theoretically motivated fixed-effect predictors), while accounting for the hierarchical nature of the data. Similarly, if researchers fit models such as CLMMs to norm items, they should either share files containing the models, or reproducible code which can be used to fit them. We encourage researchers with existing normed datasets to consider reanalysing the data with CLMMs and releasing trial-level data in addition to per-item summaries.

In addition to more accurately norming items’ central tendencies, we have shown how CLMMs can be used to explicitly model and norm latent variances, which requires the application of a Bayesian distributional modelling approach. While researchers are typically most interested in items’ central tendencies, ambiguity in judgements can also be of great theoretical relevance (e.g., Brainerd et al., 2021). We have suggested that explicitly modelling differences in latent SDs, rather than assuming equality of variance, could offer a more meaningful analogue to the traditional Likert SD reported in norming studies. This recommendation is informed by the finding that SDs of Likert ratings reflect both meaningful differences in latent variance, and artefacts of nonlinearities in response patterns. We note that when distributional CLMMs are used to disentangle meaningful and artefactual contributions to items’ SDs, the striking mean–SD relationships identified as problematic by Pollock (2018) are no longer observed. As a result, we argue that this statistical concern raised by Pollock is not inherently problematic. Rather, it reflects consequences of treating ordinal scales as continuous.

We observe, however, that the methods required to estimate Bayesian distributional CLMMs tend to be more computationally complex, and correspondingly, take substantially more time to fit. The difficulty of fitting such models may even become unfeasible for especially large datasets. Researchers may therefore wish to ignore differences in latent variance, to focus only on the perhaps more theoretically relevant estimates of latent means. In Simulation 3, we showed that assuming equality of variance in this way does not seem to reduce the accuracy of latent mean estimates to any great extent. In addition, in the final analysis of the character similarity dataset (Simpson et al., 2013), we showed that fitting a maximum likelihood model assuming homogeneity of latent variance can provide highly similar estimates of latent means to those from a Bayesian distributional model. As a result, we argue that if researchers are not interested in reporting latent variances, then simpler, equal-variance models can generally be used to estimate latent means without any great loss in accuracy. However, given that other researchers may be interested in estimates of latent SDs, the trial-level dataset should be made publicly available to allow other researchers to model such differences if they wish.

All the simulations and analyses we presented here fit models which assume a single response pattern across all observations (but which can take different shapes such as *equidistant*, *left-biased*, *right-biased*, *centre-biased*, *edge-biased*, etc.). This assumption is likely appropriate for many normed variables, as reflected in how the artefacts of response patterns are clearly observed when data is collapsed across participants (see Figs. 2, 3, and 12). However, researchers may observe that different participants, or indeed items, show distinct response patterns. In this case, a considerable degree of accuracy will be lost by failing to account for such participant- or item-related dependencies. A solution could be, rather than assuming that all participants (or items) display the same overall response pattern, to model response patterns per-participant or per-item, or both (Bolt & Johnson, 2009; Jonas & Markon, 2019). We note that CLMMs can be specified to model such variability with *brms*, by using the *thres()* term to provide participant or item IDs as a grouping variable for which thresholds should be calculated separately (e.g., response | thres(4, gr=participant_id) ~ 1 + (1|item_id) + (1|participant_id) ). Such models can be very computationally intensive, adding a large number (number of participants * number of thresholds) of parameters that need to be estimated. This is especially likely to make results from large-scale norming studies difficult to estimate (e.g., *N* = 4237 participants in Brysbaert et al., 2014). We note, however, that modelling data in such cases may be made more tractable with statistical approaches like that outlined by Selker et al. (2019), which allows an arbitrary number of thresholds to be estimated via just two parameters per participant. As a result, we recommend that researchers consider calculating thresholds separately for individual participants, although we do not evaluate the performance of such models here. An alternative could be to use a different grouping variable with fewer levels, but which accounts for differences in response patterns relatively well. As an example, it may be that differences in, say, reading skill (high, medium, low) could account for variability in participant-related response patterns such that the skill groups show distinct response patterns. In this case, calculating thresholds separately for each skill group will allow the norms to be better disentangled from response patterns, while only requiring a few more parameters to be estimated. Importantly, whether splitting estimates of threshold locations by grouping variables is appropriate, and which grouping variables it would be most appropriate to split by, will differ between norming studies and participant samples. We also note that such considerations may benefit from further investigation in future research.

Throughout the simulations and reanalysis, we have used CLMMs with probit-link functions to model Likert responses. While the probit link is convenient for estimating latent parameters more directly comparable to traditional means and SDs (as it assumes the latent variable is normally distributed), other link functions can be equally appropriate, given that the true shape of the latent distribution is usually unknown. Altering the link functions for CL(M)Ms typically results in only small changes in model parameters (McCullagh, 1980). In Simulation 4, we showed that CLMMs fit with a single link function can estimate item random effects similarly well, regardless of different violations in the assumption of the latent variable and random effect distributions. Indeed, we do not recommend any single link function for modelling rating data. If researchers wish to check that the link function they use is appropriate, they may want to fit several models to the data, using different link functions but identical formulae. Researchers could then select the model which best accounts for the data, assessed via measures of model fit such as log-likelihood. Regardless, we recommend that researchers always report the link function they used to model responses.

Similarly, all the CLMMs we presented were fit using flexible thresholds. This means that we imposed no constraints on the possible positions of the thresholds which demarcate the ordered regions of the latent distribution. An alternative would be to specify necessary features of the threshold locations, such as symmetry (around the mode of the latent distribution), or equidistance between thresholds. We consider flexible thresholds to be the most informative and most generalisable option. There may be cases when specifying constraints on threshold locations is desirable for norming items, but we argue that in such cases researchers should clearly explain and justify the use of non-flexible thresholds.

In contrast to the models examined in this paper, which focus on random effects, norming studies have frequently separated results by demographic features of participants, like gender and age (e.g., Engelthaler & Hills, 2018; Grühn & Scheibe, 2008; Kanske & Kotz, 2010; Warriner et al., 2013), or features of experimental design, such as counterbalanced order of task (e.g., Salmon et al., 2010). Similarly, researchers frequently report correlations with features of items, such as other normed or corpus-derived variables (e.g., Pexman et al., 2017, 2019; Scott et al., 2019; Stadthagen-Gonzalez & Davis, 2006; Warriner et al., 2013). While such effects could be estimated by examining correlations between relevant variables and random intercepts, we note that such variables could alternatively be incorporated into the CLMM, thereby accounting for the hierarchical variability of such effects in the random effects structure. For instance, a model estimating item norms, while also estimating the effects of age and gender of participants on ratings of individual items, could be estimated with random slopes as follows:

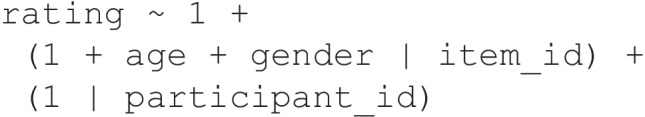



A key advantage of using CLMMs to more accurately norm items is a reduction in measurement error. As an example, consider studies examining effects of normed features of words like concreteness and imageability on behavioural or neural correlates (e.g., Goh et al., 2016; Khanna & Cortese, 2021). Such studies will be able to estimate effects more accurately, and with greater statistical power, if the normed variables more accurately reflect the underlying variable of interest, disentangled from artefacts of response patterns. Similarly, research which aims to expand the breadth of norming studies by predicting responses for unpresented items, for example via latent semantic analysis (Bestgen & Vincze, 2012), will be able to provide more accurate predictions, without simply reproducing artefacts of response patterns, if the models predict latent means rather than Likert means.

We are aware that not all norming studies employ ordinal scales. The recommendation to use CLMMs applies mostly to studies norming participant ratings, which are inherently ordinal. Some norming studies, meanwhile, norm variables which are clearly not ordinal. For example, participants may provide norms to a binomial decision, such as whether they know a given word (word prevalence; Brysbaert et al., 2019). We argue, however, that such studies can still benefit from using hierarchical modelling to pool observations and norm items more accurately. For example, random effect estimates from a binomial generalised linear mixed effects model could be used to norm word prevalence more accurately, concurrently accounting for item and participant variability, and appropriately adjusting outliers towards more accurate estimates via shrinkage. On the other hand, researchers may use scales which appear more continuous than the five-point, seven-point, and nine-point scales most commonly used in norming studies. For instance, participants may be asked to rate items on a scale from 0 to 100 (e.g., Ma et al., 2015; Yao et al., 2013). In this case, however, we argue that the only difference is in granularity; the latent continuous variable is simply separated into more regions. Participants will still show nonlinear response patterns in their judgements, biased towards some region of the scale. For such a large Likert scale there are also likely to be additional sources of nonlinearity, such as ratings biased towards numbers which are multiples of 5 or 10.

Finally, we are aware of a rich literature of existing recommendations for the formulation of Likert scales. Although such recommendations often assume the use of traditional Likert means for norming, we believe such recommendations still hold true for norming studies using the methods of analysis that we recommend here. Researchers should still carefully consider the phrasing of their questions and the instructions given to participants so as to maximise their sensitivity to the underlying variable they are interested in (Connell & Lynott, 2012; Hollis & Westbury, 2018). This will allow researchers to avoid undesirable outcomes such as floor and ceiling effects, which necessarily reduce the precision of estimates (as in Fig. 15b). Similarly, researchers should still consider whether collecting subjective judgements is informative or useful for the variable they are interested in. Regardless of how subjective judgements are analysed, they will still be inherently subjective. As an illustration, imagine a study utilising the Müller–Lyer illusion, where the sizes of lines are perceptually distorted by inward- and outward-pointing arrowheads at each end. Suppose that participants are asked to provide a Likert scale rating of how similar the two lines are in their lengths. Even if the ordinal nature of the scale is accounted for, estimates on the latent distribution will still be biased by the perceptual illusion, away from the lines’ objective lengths. This is to say, the latent variable will be disentangled from response patterns, but will inherently reflect subjective perceptions, which may not necessarily reflect objective reality.

To summarise, we have shown that CLMMs allow for much more accurate norming of items than the traditional statistics of means and SDs, which incorrectly assume the scale is continuous rather than ordinal. We argue that summarising items via estimates of their latent means and SDs provides an analogue to traditional analyses, with the advantage of appropriately disentangling variables of interest from artefacts of nonlinearities in participants’ response patterns.

## Supplementary Information

ESM 1 (DOCX 396 kb)
