# Supplementary material for: Rating norms should be calculated from cumulative link mixed effects models
Source: Behav Res Methods. 2022 Sep 14;55(5):2175–96. doi: 10.3758/s13428-022-01814-7 (PMC10439063; doi:10.3758/s13428-022-01814-7)
Supplement: Supplementary file 1 — (DOCX 396 kb) [file 13428_2022_1814_MOESM1_ESM.docx]

**Supplementary Materials 1: CLMMs offer no Additional Accuracy in Estimating Rank Order**

We conducted an additional analysis of the results of Simulation 1, to assess whether the use of raw means of Likert ratings is appropriate if researchers are only interested in the rank order of items. Here, we examined the relationship between the rank positions of each iteration’s simulated latent mean, and the estimate of its rank position from (A) raw means, and (B) estimated latent means (**Figure S1**). This revealed that, indeed, rank order is relatively unaffected by differences in response patterns, and CLMMs offer no additional gain in accuracy of estimating rank positions. However, as with any continuous variable, we note that ranking considerably increases noise in the relative distances between items. This is because a rank difference of 1 could be a very large difference in the original units if at a position where items are sparsely spread, or a very small difference if at a point where items are densely clustered. Therefore, when researchers are interested in the relative distances between items, we recommend the usage of ordinal models like CLMMs to appropriately account for the ordinal nature of Likert scales.


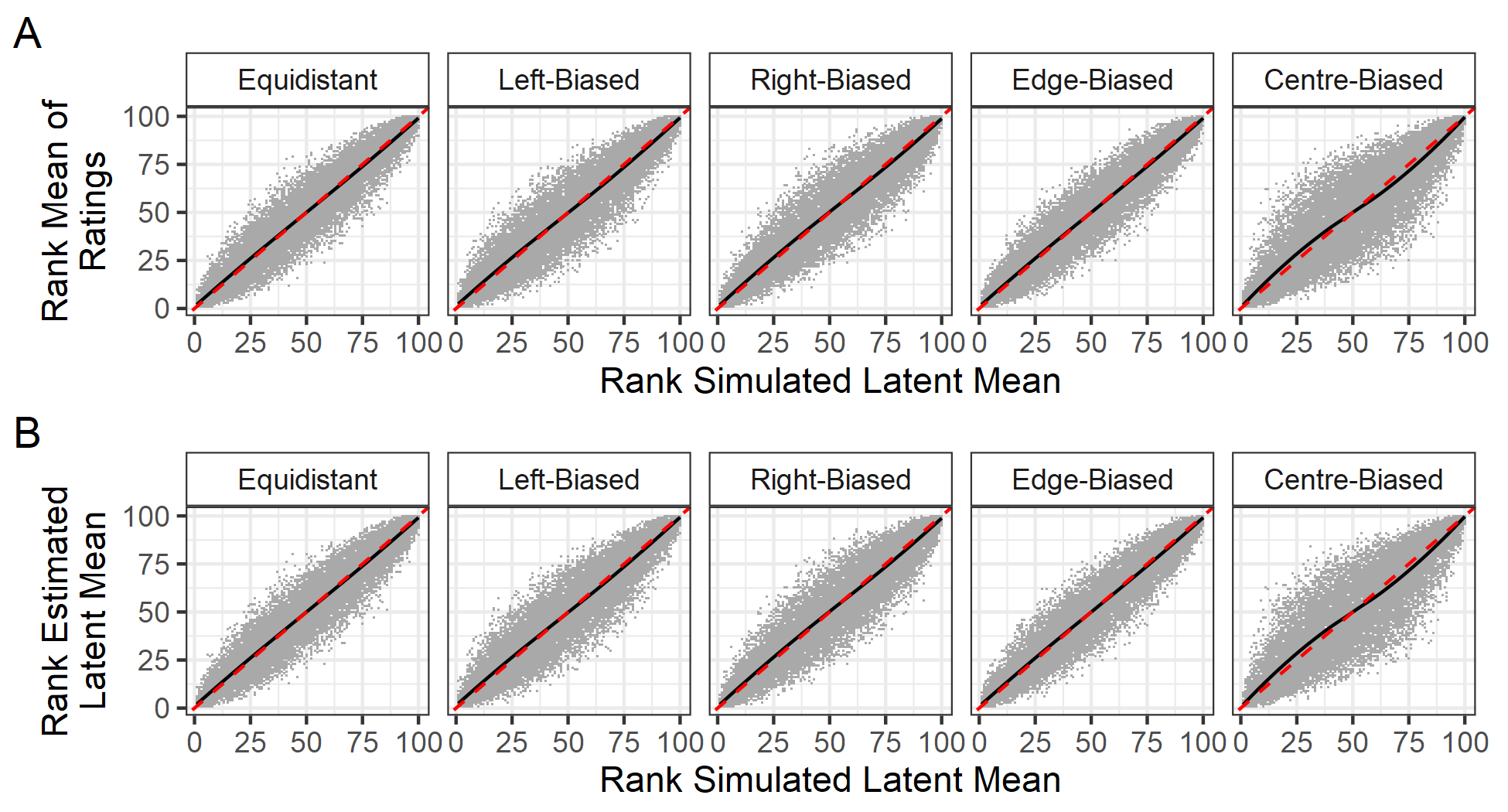


**Figure S1.** The relationship between rank simulated latent means, and (A) rank raw means, or (B) rank estimated latent means (from CLMMs). Grey points show the results for individual items (ranked within their iterations). The relationships shown with the black lines were estimated via locally estimated scatterplot smoothing (LOESS), with a span parameter of .75. The dashed red lines show an expected linear relationship for reference, identical across all response patterns.

**Supplementary Materials 2: Within-Participant *Z*-Scores of Raw Responses do not Account for Ordinal Nature of Likert Responses**

One approach which researchers may consider when norming items on Likert ratings, where responses are provided by multiple participants, is to firstly *z*-score responses within each participant. However, we argue that such an approach still fails to account for the ordinal nature of Likert ratings, and consequently still entails the distortion in norming estimates which we identify for averages of raw responses.

A possible justification for *z­*-scoring responses within participants may be that per-item averages should be less biased by individual participants’ response styles. For instance, participants who consistently respond with extreme (i.e., very high or low) ratings, will exhibit correspondingly extreme averages and low *SD*s. Within-participant *z*-scores for such a participant would thus align, more closely than raw responses would, with the *z*-scores of participants who responded with ratings less extreme and more variable. However, this approach still assumes that the raw Likert responses are continuous, rather than ordinal. In this way, researchers applying this approach to norming items should still expect the norms to be distorted by nonlinear response styles. To demonstrate this, we re-analysed the results from Simulation 2, comparing the performance of the CLMM approach to that of within-participant *z*-scores in norming items. The results (**Figure S2**) showed that in assuming responses are continuous, the *z*-scoring approach results in a distortion of item norms very similar to that observed for averages of raw responses.


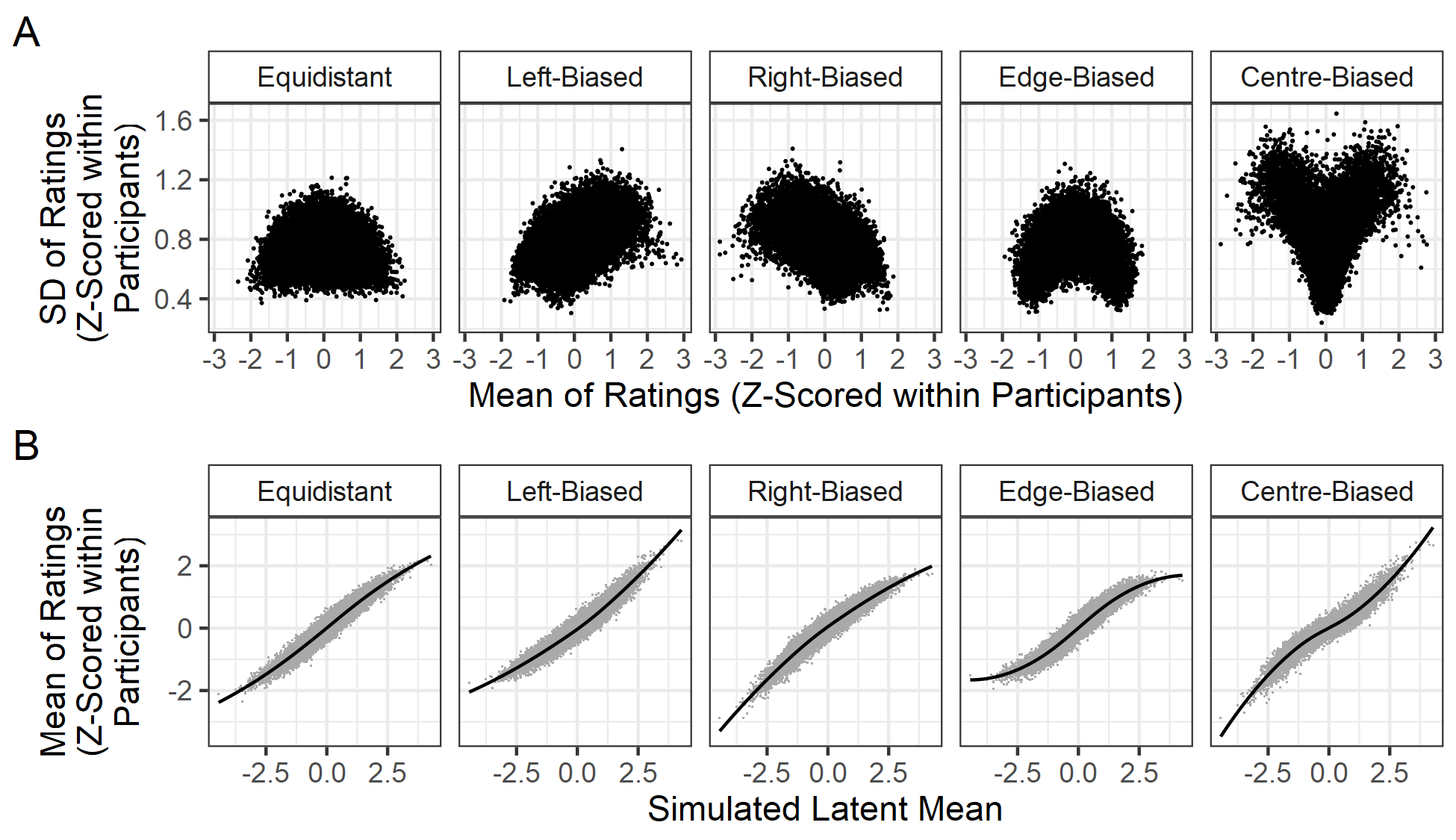


**Figure S2.** Results of the within-participant *z*-scoring analysis of Simulation 2: The *z*-scoring approach results in similar patterns of distortion to those observed for raw means. The panels show (A) the relationship between means and *SDs* of ratings, and (B) the relationship between items’ simulated latent means and mean rating. The relationships shown with the black lines were estimated via LOESS, with a span parameter of .75.

The rationale behind *z*-scoring responses within participants, namely that *raw* means of ratings will be biased by variability between participants, is well-considered. Nevertheless, we argue that the random effects structures of CLMMs are better placed to account for between-participant variability in ordinal models, as they estimate these differences in the latent distribution, rather than assuming raw responses scale linearly. Furthermore, accounting for participant variability via random effects allows the impact of item and participant variability to be estimated simultaneously, and thus more accurately. This is preferable to the separate steps (i.e., *z*-score within participants, *then* calculate per-item averages) of the *z*-scoring approach. As an example, consider a design where participants each rate only a small random subset of a pool of items: here, it is likely for a single participant to be presented with items that all happen to be extremely high or low in the feature which is being rated. Accounting for participant variability in a separate step before calculating item norms would result in such a participant’s ratings being adjusted away from the factually extreme responses, thereby reducing the magnitude of the average ratings for the items they rated. Item and participant random effects estimated in a single model, in contrast, would more accurately reflect both sources of variability. To use the example again, the ratings of participants who were presented with only extreme items would align with the ratings provided by participants who were *not* presented with such a biased sample of items. By estimating both sets of random effects simultaneously, the partially pooled model would be able to disentangle item and participant variability, without requiring each participant to be presented with a necessarily representative sample of items. Finally, we again note that pooling of the data allows the random effects structure to confer additional accuracy via shrinkage, where unlikely extreme observations are appropriately adjusted towards more likely estimates.
